# Elucidation of Triacylglycerol Overproduction in the C_4_ Bioenergy Crop *Sorghum bicolor* by Constraint-Based Analysis

**DOI:** 10.3389/fpls.2022.787265

**Published:** 2022-02-17

**Authors:** Teresa J. Clark, Jorg Schwender

**Affiliations:** ^1^Biology Department, Brookhaven National Laboratory, Upton, NY, United States; ^2^Department of Energy Center for Advanced Bioenergy and Bioproducts Innovation, Upton, NY, United States

**Keywords:** bioenergy grasses, metabolic reconstruction, C_4_ photosynthesis, energy balance, plant lipids, triacylglycerol, constraint-based analysis, *Sorghum bicolor*

## Abstract

Upregulation of triacylglycerols (TAGs) in vegetative plant tissues such as leaves has the potential to drastically increase the energy density and biomass yield of bioenergy crops. In this context, constraint-based analysis has the promise to improve metabolic engineering strategies. Here we present a core metabolism model for the C_4_ biomass crop *Sorghum bicolor* (*iTJC1414*) along with a minimal model for photosynthetic CO_2_ assimilation, sucrose and TAG biosynthesis in C_3_ plants. Extending *iTJC1414* to a four-cell diel model we simulate C_4_ photosynthesis in mature leaves with the principal photo-assimilatory product being replaced by TAG produced at different levels. Independent of specific pathways and per unit carbon assimilated, energy content and biosynthetic demands in reducing equivalents are about 1.3 to 1.4 times higher for TAG than for sucrose. For plant generic pathways, ATP- and NADPH-demands per CO_2_ assimilated are higher by 1.3- and 1.5-fold, respectively. If the photosynthetic supply in ATP and NADPH in *iTJC1414* is adjusted to be balanced for sucrose as the sole photo-assimilatory product, overproduction of TAG is predicted to cause a substantial surplus in photosynthetic ATP. This means that if TAG synthesis was the sole photo-assimilatory process, there could be an energy imbalance that might impede the process. Adjusting *iTJC1414* to a photo-assimilatory rate that approximates field conditions, we predict possible daily rates of TAG accumulation, dependent on varying ratios of carbon partitioning between exported assimilates and accumulated oil droplets (TAG, oleosin) and in dependence of activation of futile cycles of TAG synthesis and degradation. We find that, based on the capacity of leaves for photosynthetic synthesis of exported assimilates, mature leaves should be able to reach a 20% level of TAG per dry weight within one month if only 5% of the photosynthetic net assimilation can be allocated into oil droplets. From this we conclude that high TAG levels should be achievable if TAG synthesis is induced only during a final phase of the plant life cycle.

## Introduction

Plant oils are valuable crop products because of their energy density and potential biofuel use. While plants accumulate oils mostly as triacylglycerol (TAG) in specialized organs, there is increasing interest in developing bioenergy crops that accumulate TAG in the vegetative parts of the plant (Durrett et al., 2008; Shih et al., 2016; Xu and Shanklin, 2016; Lee et al., 2017). While oilseeds can accumulate between 20 and 50% TAG by weight (Ohlrogge and Chapman, 2011; Wan et al., 2017), seeds typically make up only a fraction of the plant biomass produced during a growing cycle. It has been estimated that if the bulk of above ground harvested plant biomass would contain TAG at 10% (w/dw), yields per acre could be substantially higher than achievable for any seed oil crop (Ohlrogge and Chapman, 2011). Also, as a by-product, vegetative TAG would be particularly beneficial in high yielding bioenergy grasses that are already harvested for other compounds (Carpita and McCann, 2008; Weijde et al., 2013). Sugarcane, for example, has been engineered to accumulate on average 4.3% (w/dw) TAG in its stems and up to 8% (w/dw) TAG in its leaves (Parajuli et al., 2020) and a techno-economic analysis determined that processing sugarcane containing 5% TAG per total dry weight for biodiesel in addition to converting its native sugars into ethanol would be economically advantageous, and 20% TAG could nearly double the potential profits compared to normal sugarcane (Huang et al., 2016).

However, vegetative tissues like leaves tend to have low intrinsic capacity to produce and store TAG at high levels (Chapman et al., 2013; Xu and Shanklin, 2016). It is therefore to be expected that efforts to engineer accumulation of TAG in vegetative tissues requires complex reprogramming of metabolism. To date, the most successful studies on engineering plants to accumulate TAG in vegetative tissues have used combinatorial gene expression approaches and many of the applied designs can be related to the “push-pull-protect” paradigm (Vanhercke et al., 2014, 2019b). Here the general strategy implies that substantial TAG accumulation requires the manipulation of multiple gene targets to (1) divert a portion of carbon fixed by primary photosynthesis into fatty acid synthesis (“push”), (2) increase the capacity to assemble *de novo* synthesized fatty acids into TAG (“pull”), and (3) suppress leaf intrinsic lipid degradation processes (“protect”) that frequently appear to counteract TAG accumulation (Chapman et al., 2013; Xu and Shanklin, 2016; Vanhercke et al., 2019b). In a recent survey of more than 20 studies aimed at maximizing TAG accumulation in vegetative plant tissues (Vanhercke et al., 2019b), the median of the reported TAG levels is 3.6% (w/dw), with the highest levels reaching close to 30% (w/dw) in leaves of *Nicotiana tabacum* and *Nicotiana benthamiana*. As for possible limitations to the accumulation of TAG in vegetative tissue, the high biosynthetic cost of lipid synthesis comes to mind. TAG is a highly reduced form of carbon with more than twofold the energy density of protein or carbohydrate (Ohlrogge and Chapman, 2011). Considering TAG as an alternative carbon sink to sucrose, the principal product of leaf photosynthesis, it is of interest to know how much additional metabolic cost is incurred if TAG is synthesized as the alternative photo-assimilatory product and at which level of TAG accumulation the overall carbon and energy balance of a plant can be expected to be substantially affected. Several studies aimed at engineering vegetative TAG accumulation reported yield penalty effects (Kim et al., 2015; Vanhercke et al., 2017; Chu et al., 2020; Mitchell et al., 2020; Parajuli et al., 2020) which might be attributed to the high biosynthetic cost of TAG, or to energy losses caused by futile cycles of lipid synthesis and degradation. However, there also have been reports of overall increased photosynthetic capacity and plant growth in TAG over-accumulating *Arabidopsis thaliana* and *Lolium perenne* (Winichayakul et al., 2013; Beechey-Gradwell et al., 2020; Cooney et al., 2021). It appears that we currently lack a good understanding of the effects of TAG over-accumulation on metabolism, physiology, and the overall plant life cycle. Studies on vegetative TAG engineering generally report TAG levels in tissues as metabolic end points, without assessing *in vivo* metabolic rates or defining a theoretical yield as a benchmark. It therefore would be useful to explore the theoretical capacity of a green vegetative tissue to accumulate TAG based on the capacity to photo-assimilate CO_2_. Assuming that the metabolic network can be manipulated as to divert intermediates of the Calvin–Benson–Bassham (CBB) cycle away from synthesis of sucrose and other photo-assimilates toward TAG synthesis, the achievable TAG accumulation rate will depend on how much of the fixed carbon can be re-allocated and on the cost in energy cofactors for TAG synthesis.

Toward that end, here we present a genome-referenced core metabolic model (*iTJC1414*) of *Sorghum bicolor*, a high biomass yielding C_4_ crop species, with detailed manual curation of NADP-malic enzyme (NADP-ME) type C_4_ photosynthesis, lipid metabolism and other parts of central metabolism. To investigate the metabolic potential to photo-assimilate CO_2_ into carbohydrate or TAG, we constructed a two-cell leaf model representing C_4_ metabolism (*iTJC1414x2*) that was further expanded into a diel model that simulates cycles of day and night leaf metabolism (*iTJC1414x4*). To assess the potential for TAG biosynthesis, we compared the chemical balances and biochemical pathways for assimilation of CO_2_ into sucrose and TAG, respectively. We analyzed the costs and the supply/demand balance between photosynthetic supply and biosynthetic demands of energy cofactors (ATP and NADPH). Using *iTJC1414x4*, we simulated partitioning between the phloem-exported photo-assimilates and accumulated oil droplets and predicted their potential daily net productions. A series of scenarios was used to predict how fast TAG could accumulate in a mature leaf, dependent on the fraction of assimilated carbon assumed to be diverted into TAG accumulation. We further assessed the effect of metabolic futile cycles of TAG or fatty acid biosynthesis and degradation in draining cellular energy reserves and limiting TAG accumulation. Overall, our findings reveal that based on the typical photosynthetic potential of a sorghum leaf, TAG could accumulate much faster than the typical duration of the life cycle. If only 5% of the primary carbon fixation is diverted into TAG synthesis without superimposed futile cycles, a 20% yield target (TAG weight per leaf dry weight) could be reached within only 18 simulated day/night cycles. Our quantitative assessments give a perspective on past efforts at metabolic engineering of TAG accumulation in photosynthetic plant tissues as well as might serve as a guide for future efforts in this direction.

## Materials and Methods

### Reconstruction of *Sorghum bicolor* Metabolic Model

In the following we describe the reconstruction of an *S. bicolor* four-cell metabolic model (*iTJC1414x4*) with the capacity to simulate C_4_ photosynthesis as well as to integrate a day phase with a night phase during which storage compounds accumulated during the day are consumed. We based the metabolic reaction network on *iEB2140x2* (Bogart and Myers, 2016), a two-cell high confidence representation of *Zea mays* core metabolism with 635 reactions of which 469 are associated to a total of 2,140 maize genes. To relate the model to *S. bicolor*, we derived genome wide synteny/orthology associations between maize and sorghum. Predicted protein sequences for amino acid sequences and chromosomal order of 63,480 *Z. mays* protein encoding genes (B73 v3, Schnable et al., 2009) and of 34,129 *S. bicolor* protein encoding genes (*S. bicolor* cultivar BTx623, genome assembly v3.1.1, McCormick et al., 2018) were obtained from Phytozome genomic resource.^1^ *Z. mays* protein sequences were used as queries to identify orthologs in *S. bicolor* using the SynOrths tool (version 1.0, Cheng et al., 2012). In short, this tool identifies likely pairs of ortholog genes in two related species based on similarities in protein sequences with additional support from homologous flanking genes. For *Z. mays* genes for which SynOrths did not predict orthologs, protein BLAST (version 2.8.1, Altschul et al., 1997) was used to align amino acid sequences and top hits were assumed to be orthologs if they had at least 90% alignment and 70% identity. Since maize is tetraploid (Swigonova et al., 2004) while *S. bicolor* is diploid (Price et al., 2005), we expect quite frequently two or more maize genes to map to the same sorghum locus. Indeed, out of over 24,000 *S. bicolor* genes for which orthology relations were identified, in more than 60% of cases, an *S. bicolor* gene was orthologous to more than one *Z. mays* gene (Supplementary Figure 1A).

The *Z. mays* modeling file (*iEB2140x2*) was obtained from Bogart and Myers (2016) and transferred to *S. bicolor* in a spreadsheet format (COBRA toolbox, Microsoft Excel 2016). The model was extended from a two-cell model (x2) connecting a bundle-sheath cell (BSC) and a mesophyll cell (MC) to a diel model (x4). Per sub-model, reactions were expanded or validated using the Kyoto Encyclopedia of Genes and Genomes,^2^ Plant Metabolic Pathway Databases,^3^ and Phytozome v12.1. The network reactions were revised according to literature, as presented in Supplementary Table 1. Briefly, we moved the reactions for *de novo* fatty acid biosynthesis (through C_18_) from the cytosol to the plastid. Acetyl-coenzyme A (CoA) in the plastid can be diverted directly from the CBB cycle by the C_3_→C_2_ pathway, involving conversion of 3-phosphoglycerate (3-PGA) *via* 2-PGA, phosphoenolpyruvate and pyruvate to acetyl-CoA (Joyard et al., 2010). TAG was not represented in the initial maize model, so we added its synthesis from phosphatidylcholine (PC) and/or diacylglycerol (DAG), as well as its breakdown into glycerol and free fatty acids. The lipid classes were originally represented by generic molecules (Bogart and Myers, 2016). To simulate metabolic effects resulting from differences in fatty acid chain lengths, we took an approach similar to that taken by Simons et al. (2014) and defined four majorly abundant fatty acid species (C16:0, C18:1, C18:2, and C18:3) to be associated with cytosolic PC, DAG, and TAG molecular species without assigning stereospecific positioning (e.g., *sn*-1 and *sn*-2). We added reactions to synthesize these lipid classes, expanded how PC-bound fatty acids are desaturated by cytosol/endoplasmic reticulum-localized desaturase enzymes, and added a PC-dependent acyl editing cycle (Li-Beisson et al., 2013). Next, to allow for testing the effects of futile cycles of lipid synthesis and degradation, we added peroxisomal β-oxidation to recycle cytosolic free fatty acids as well as CoA ligation reactions (Long-Chain Acyl-CoA Synthetase, EC 6.2.1.3) to reuse free fatty acids to synthesize TAG.

For numerical simulations the model was converted to Systems Biology Markup Language (SBML) using a Python^4^ script derived from Hay et al. (2014). Linear programming analyses were performed using the constraint-based reconstruction and analysis (COBRA) toolbox version 3.1 (Heirendt et al., 2019) and GLPK solver^5^ within the MATLAB R2018b environment (The MathWorks, Natick, MA, United States). Mathematical flux ratio constraints were imposed using the COBRA function “addCOBRAConstraints.” The *S. bicolor iTJC1414* single-cell model is provided in Supplementary File 1. The full leaf diel *iTJC1414x4* model with simulation codes are provided in Supplementary File 2.

### Model Constraints for the NADP-Malic Enzyme Subtype of C_4_ Photosynthesis

**FIGURE 1:**
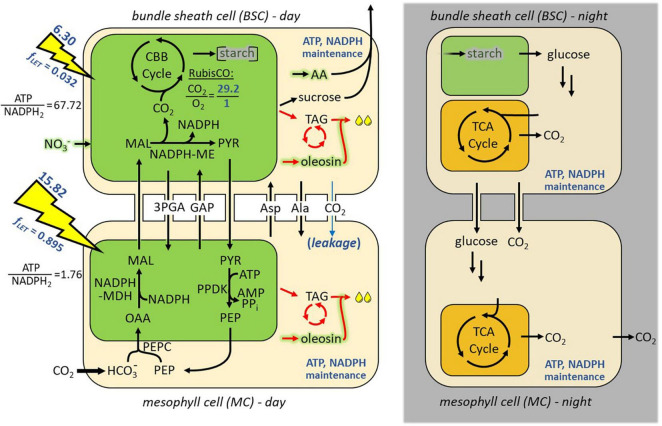
Diel model of NADP-ME subtype C_4_ photosynthesis in mesophyll and bundle sheath cells (*iTJC1414x4*). Shown are the major metabolic processes in mesophyll (bottom row) and bundle sheath (top row) cells that are predicted to take place during photo-assimilation. Chloroplastic and mitochondrial compartments are shown in green and orange, respectively. Model constraints that pertain to the mature leaf reference state described in the text (i.e., distribution of photosynthetic energy, maintenance respiratory processes, RubisCO oxygenation, and CO_2_ leakage) are indicated in blue (see also Supplementary Table 2). For both cell types, fixed proportions of ATP and NADPH supply by light reactions are indicated (see main text). Carbon reallocation to TAG and lipid futile cycle pathways is are indicated in red (see main text). Diel cycling was simulated with light being only accessible for the day sub-models (left column), which provides starch for use by the night sub-models (right column). On the night side of the model catabolic and respiratory activity is caused by fixed ATP and NADPH maintenance costs. Nitrate assimilation is indicated in green shading. 3PGA, 3-phosphoglycerate; AA, amino acids; AcCoA, acetyl coenzyme A; Ala, alanine; Asp, aspartate; CBB cycle, Calvin–Benson–Bassham cycle; *f*_*LET*_, fraction of absorbed light that drives linear electron transport; GAP, D-glyceraldehyde 3-phosphate; MAL, malate; ME, malic enzyme; MDH, malate dehydrogenase; NO_3_^–^, nitrate; OAA, oxaloacetate; PEP, phosphoenolpyruvate; PEPC, phosphoenolpyruvate carboxylase; PPDK, pyruvate phosphate dikinase; PYR, pyruvate; RubisCO, ribulose 1,5-bis-phosphate carboxylase/oxygenase; TAG, triacylglycerol; TCA, tricarboxylic acid.

Several numerical constraints were added to the model for model simulations to be consistent with the NADP-ME subtype of C_4_ photosynthesis (Supplementary Table 2). In plants with the NADP-ME subtype, the malate/pyruvate transfer is the main mechanism to shuttle CO_2_ from MCs to BSCs, while a secondary aspartate/malate shuttle mechanism has been reported to be of significance as well in NADP-ME C_4_ plants like sorghum (Yin and Struik, 2018). These transport flows through plasmodesmata are generally thought to be driven by strong concentration gradients between the cell types (Wang et al., 2014a; Arrivault et al., 2017), which means that malate, for example, can be assumed to be always moving from MCs to BSCs and never in the opposite direction. Accordingly, among the plasmodesmata transport reactions available in *iTJC1414x4*, we allowed transport of malate and aspartate from MCs toward BSCs as well as pyruvate and alanine in the opposite direction (Figure 1 and Supplementary Table 2). NADP-ME type species tend to contain little capacity for photoreduction of 3-PGA in the BSCs (Hatch, 1987), which suggests the operation of a 3-PGA/triose phosphate shuttle between BSCs and MCs so that part of the 3-PGA reduction can take place in the MCs. Accordingly, we allowed 3-PGA and glyceraldehyde 3-phosphate to move between the cell types. Due to the presence of various isoforms of ME in the model, cytosolic isoforms and the plastidic NADH-dependent isoform were inactivated so that only the BSC plastidic NADP-ME will be active in decarboxylation of malate in the C_4_ cycle. Phosphoenolpyruvate carboxykinase is another decarboxylating enzyme in C_4_ metabolism known to be of relevance in species of the NADP-ME subtype. However, the reaction was inactivated in our model because phosphoenolpyruvate carboxykinase activity was reported to be missing in sorghum (Gutierrez et al., 1974; Walker et al., 1997; Döring et al., 2016). Some CO_2_ liberated by decarboxylation of malate is expected to leak from BSCs to the MCs due to the concentration gradient between the two cells (von Caemmerer and Furbank, 2003; de Oliveira Dal’Molin et al., 2010; Wang et al., 2014b). Jenkins et al. (1989) estimated this leakage to comprise 13.4% of the inorganic CO_2_ liberated by C_4_ acid decarboxylation in the BSCs. Since essentially all the CO_2_ fixed in BSCs passes through ribulose bisphosphate carboxylase/oxygenase (RubisCO), we applied the 13% leakage estimate on the rate of RubisCO carboxylation. As well, from the expected CO_2_ and O_2_ availabilities in BSCs, RubisCO carboxylase activity is expected to be 29.2-fold greater than its oxygenase activity in NADP-ME-type C_4_ plants (Jenkins et al., 1989). Consequently, the CO_2_ leakage and photorespiration reactions were constrained to match these literature estimates (Supplementary Table 2).

### Modeling of Diel Metabolism

CO_2_ assimilation by C_4_ photosynthesis and allocation of reduced carbon was modeled by considering the interactions of day and night metabolism in a diel Flux Balance Analysis (FBA) model similar to other reported diel plant models (Cheung et al., 2014; de Oliveira Dal’Molin et al., 2018; Shaw and Cheung, 2018). In essence, the *S. bicolor* C_4_ core metabolism model with interconnected MC and BSC sub-models (*iTJC1414x2*) was duplicated into representations of day and night. In the resulting diel model with four cellular sub-models (*iTJC1414x4*), the BSC day and night cells share a common pool that represents transitory starch (Figure 1), while MCs are assumed to not store starch. This is according to literature findings that starch is observed mainly in the BS chloroplasts of C_4_ species like *Z. mays* and sorghum (Lunn and Furbank, 1997). Light is only available as an energy source for the day sub-models which contribute to the starch pool while the night models rely on starch degradation to fuel dark metabolism and respiration (Supplementary Table 2). In general, flux states were obtained based on the principle of light-limiting conditions. Given a fixed biosynthetic rate of a photosynthetic product, total light uptake fluxes were minimized. Alternatively, given fixed light uptake fluxes, the biosynthetic rate of a photosynthetic product was maximized. To simulate C_4_ photosynthesis, only the MC is allowed to exchange CO_2_ with the external environment and RubisCO is only active in the BSC (Supplementary Table 2). Net CO_2_ uptake (*V*_*netCO2*_) is defined as the difference between daily uptake of CO_2_ (*V*_*CO2uptake*_) and CO_2_ loss at night (*V*_*CO2export*_):


(1) $$V_{n\,e\,t\,C\,O\,2}=V_{C\,O\,2\,u\,p\,t\,a\,k\,e}-V_{C\,O\,2\,e\,x\,p\,o\,r\,t}.$$

By linking the four sub-models (BSC-day, MC-day, BSC-night, and MC-night) through the shared starch pool, a single linear optimization procedure predicts fluxes that are in steady-state over the integrated day-night cycle.

### Simulating Carbon Allocation

Model analyses in this study assume cells are mature, thus no longer growing, and TAG and sucrose are the primary net carbon sinks. Although we modeled non-growing cells in this study, we also updated the *iTJC1414x4* biomass composition constraints using published *S. bicolor* carbohydrate, lignin, amino acid, and lipid ratios (Supplementary Table 3) and confirmed that the model was functional if maximizing total biomass was used as the objective function. The fatty acid composition of TAG was defined as reported for wild-type sorghum leaves (Vanhercke et al., 2019a). Transgenic studies aimed at increasing TAG accumulation in vegetative tissues have regularly reported TAG compositions that are distinctly different from the wild-type (Xu and Shanklin, 2016; Vanhercke et al., 2019b). As described in section “Results,” changes in TAG fatty acid composition that have been observed in sorghum high-oil lines (Vanhercke et al., 2019a) do not majorly affect biosynthetic energy demands and were therefore not considered here. Similarly, we did not consider here the possibility that increased leaf TAG levels may in part be attributable to re-balancing fatty acids between membrane lipids and TAG. For example, in a recent study on high oil accumulating tobacco lines (Zhou et al., 2020) there was a dramatic decrease in levels of chloroplastic galactolipids in the transgenic line which likely benefited TAG production. Our model does not consider the potentially involved lipid trafficking mechanisms between chloroplast and cytosol/endoplasmic reticulum. To our knowledge these mechanisms are not yet fully elucidated. We verified that sucrose and precursors for TAG biosynthesis can be generated by canonical pathways. In short, the primary CO_2_ fixation product in the CBB-cycle in the chloroplast, 3-PGA, can be directly transformed into pyruvate by a series of glycolytic reactions, which can then be transformed into acetyl-CoA by the plastid-localized pyruvate dehydrogenase complex (PDHp) (Bassham and Calvin, 1960; Li-Beisson et al., 2013). Alternatively, PGA can be reduced to triose phosphates (Bassham and Calvin, 1960), which are then transformed into transitory starch or sucrose for export (Raines, 2003).

For modeling carbon allocation scenarios, we consider here that the primary products of photo-assimilation are exported *via* phloem to other organs or that, due to an envisioned transgenic intervention to engineer TAG accumulation, a part of the assimilated carbon is diverted into oil droplets and stored in the leaf. Apart from changes in leaf TAG levels, the carbon allocation scenarios simulated here do not consider changes in leaf levels of other biomass constituents such as sugars, starch, or amino acids. While for a mature leaf, sucrose can be understood as the predominant exported photo-assimilate, it can be considered that amino acids are exported as well, which is supported by studies of phloem sap composition in various plants (Wilkinson and Douglas, 2003). We therefore defined photo-assimilate based on the composition of sucrose and amino acids found in phloem sap of barley leaves (Winter et al., 1992). The combined assimilate export is comprised of sucrose and 17 amino acids (Supplementary Table 4) with a sucrose to amino acid ratio of 94:6 (by weight). Also, while TAG can be considered to be the predominant product of the envisioned metabolic engineering effort, oleosin proteins are major components of oil droplets that are stored as intracellular particles (Ohlrogge and Chapman, 2011). Oleosins were estimated to comprise 1–5% of the oil droplet weight in maize embryos while the remainder is mostly TAG (Ting et al., 1996). Consequently, in carbon allocation simulations we define the deposition of oil droplets as biosynthesis of TAG and oleosin at a 95:5 ratio (by weight) (Supplementary Table 5). Here, the oleosin has an amino acid composition that matches *Sesamum indicum* oleosin-L (Tai et al., 2002; Supplementary Table 5). This protein was over-expressed in sorghum leaves in the study of Vanhercke et al. (2019a).

Note that nitrate reduction and ammonia assimilation into amino acids take place in the simulated assimilate export as well as in oleosin synthesis. Nevertheless, since nitrogen plays only a minor role, we will keep here the term “carbon allocation” for the model simulations of assimilate export, and TAG and oleosin deposition. For simulations on TAG accumulation, the objective function used was to maximize the TAG and oleosin accumulation in MC and BSC during the day. This allows TAG and oleosin biosynthesis to take place in day metabolism for both cell types. This procedure appears to be justified because when WRI1, DGAT, and oleosin were expressed under a constitutive promoter in sorghum, oil droplets were found to accumulate in both cell types (Vanhercke et al., 2019a). For simulations of carbon allocation scenarios, different carbon allocation ratios were enforced based on the equation


(2) $$P_{o\,i\,l}=\left(1-\frac{12.77162\times V_{A\,e\,x}}{V_{n\,e\,t\,C\,O\,2}}\right)\times 100\%,$$

where *P*_*oil*_ is the percentage of net CO_2_ uptake transformed into TAG and oleosin, and *V*_*Aex*_ is the assimilate export flux to the phloem, with the average photo-assimilate being 12.77162 mol carbon per mol. For any chosen carbon allocation ratio *P*_*oil*_ > 0%, the model can be optimized by maximization of the total oil droplet accumulation rate. Because steady-state day-night cycling is integrated into the model, the simulations predict fluxes in units of mol/m^2^/day, including the total accumulation rate of TAG.

Given a daily oil droplet accumulation rate (*V*_*oilDroplet*_), one can determine the time period needed (*t*) in days for a full-grown leaf to accumulate a desired amount of TAG per dry weight:


(3) $$t=\frac{C_{T\,A\,G}\times L}{\left(100\%-C_{T\,A\,G}\right)\times M_{T\,A\,G}}\times \frac{1}{V_{o\,i\,l\,D\,r\,o\,p\,l\,e\,t}},$$

where *C*_*TAG*_ is the desired TAG content in the leaf expressed as percent dry weight, *L* is the initial dry weight per leaf area which was set to 60 g/m^2^, as reported for sorghum (Zhao et al., 2005). *M*_*TAG*_ is the molecular weight of TAG (866.811 g/mol) based on the molecule species composition as defined in the model biosynthesis reactions. Note that the reaction equation for the daily oil droplet accumulation rate (*V*_*oilDroplet*_) is defined so that 1 mol TAG is produced (866.81 g) along with 45.55 g oleosin, so that oleosin is 5% of the total in weight (Supplementary Table 5).

### Testing Futile Lipid Cycles

We used our model to explicitly test two types of futile cycles that will be referred to as TAG cycling and FA cycling. TAG cycling is defined as the ratio of fluxes toward TAG synthesis and TAG storage in oil droplets (detailed equations in Supplementary Table 6):


(4) $$\mathrm{TAG}\,\mathrm{cycling}=\frac{V_{T\,A\,G\,s\,y\,n\,t\,h\,e\,s\,i\,s}}{V_{o\,i\,l\,D\,r\,o\,p\,l\,e\,t}}.$$

Succinctly, the TAG cycling ratio indicates to what extent TAG is over-produced for the predicted rate of TAG storage. The excess TAG must be hydrolyzed into glycerol and free fatty acids. As will be seen from the model simulations (see section “Results”), under limited energy input (light) and with increasing TAG cycling there will be reduced flux into TAG storage. Like TAG cycling, FA cycling is the ratio of fluxes toward fatty acid synthesis compared to that which is used for TAG storage in oil droplets (detailed equations in Supplementary Table 6):


(5) $$\mathrm{FA}\,\mathrm{cycling}=\frac{V_{F\,A\,s\,y\,n\,t\,h\,e\,s\,i\,s}}{3\times V_{o\,i\,l\,D\,r\,o\,p\,l\,e\,t}}.$$

By defining FA cycling in this way, the over-produced portion of fatty acids cannot be utilized and is therefore subjected to degradation (β-oxidation). Products and energy cofactors generated by the degradation process are recycled.

### Defining Photosynthetic Energy Cofactor Production

The default configuration of *iTJC1414x4* allows photon flux to freely distribute between the BSC and MC and between the linear and cyclic components of photosynthetic electron transport. To model the light absorption more realistically, we generated sorghum-specific light flux distribution settings from a model for cell-type specific electron transport in C_4_ photosynthesis (Yin and Struik, 2018), which integrates multiple photosynthesis-related biophysical and biochemical characteristics (see Supplementary File 3). One predicted parameter, *a*_*BS,M*_, defines the ratio at which total received light absorptance is partitioned between the BSC and MC (Yin and Struik, 2018). This ratio was applied to *iTJC1414x4* as a linear dependency between the two light uptake fluxes:


(6) $$m\,s\,D\,a\,y\,t\,x\,L\,i\,g\,h\,t\times a_{B\,S,M}-b\,s\,D\,a\,y\,t\,x\,L\,i\,g\,h\,t=0.$$

The model by Yin and Struik (2018) also predicts the fraction of the absorbed light that drives linear electron transport (LET) for each of the two cell types. Here we designate these as *f*_*LET,M*_ and *f*_*LET,BS*_ for the MC and BSC, respectively. To add the *f*_*LET*_ parameters as numerical constraints to *iTJC1414x4* we derived the following equation (for details see Supplementary File 3):


(7) $$1.0625\times v_{P\,S\,I}\times f_{L\,E\,T}+v_{P\,S\,I\,I}\times \left(5\times f_{L\,E\,T}-9.25\right)=0,$$

with *v*_*PSI*_ and *v*_*PSII*_ being the rates of the reactions representing photosystem I and II in *iTJC1414x4*, respectively (“*PhotosystemImodchloroplast*,” “*PhotosystemIImodchloroplast*”). Applying the energy budget parameters *a*_*BS,M*_, *f*_*LET,BS*_ and *f*_*LET,M*_ to *iTJC1414x4* by Equations 6, 7 will cause the photosynthetic supply of ATP and NADPH to be at fixed proportions in the BSC and MC and at a fixed overall ATP/NADPH supply ratio. If the photosynthetic supply is fixed and the biosynthetic output has a fixed composition, there can be an imbalance between supply and metabolic demands of ATP and NADPH. Such an imbalance causes one of the two energy cofactors to be overproduced, i.e., it will either accumulate or dissipate in a process that is not related to biosynthesis. In the context of *iTJC1414x4*, no accumulation of metabolites can materialize since FBA is a steady-state modeling approach. Instead, we characterize imbalanced scenarios in *iTJC1414x4* by detecting net hydrolysis of ATP that does not drive biosynthesis and by detecting mitochondrial oxidative phosphorylation that leads to more ATP production at the expense of oxidation of photosynthetically generated reducing equivalents. Specifically, ATP surplus is quantified by maximizing an ATP-consuming dummy reaction in a secondary optimization (Supplementary Figure 2). The size of the flux through complex IV of the mitochondrial electron transport chain (“*ComplexIVmodmitochondrion*”) reveals the amount of photosynthetically produced reducing equivalents that are transferred back to oxygen. Both ATP surplus and NADPH surplus are quantified by a Flux Variability procedure as indicated in Supplementary Figure 2. The ATP surplus and NADPH surplus quantified this way reveals misalignment between photosynthetic supply of ATP and NADPH and biosynthetic demands. Tests showed that if the energy budget parameters are not applied, no ATP or NADPH surplus is detected. Furthermore, as detailed in Supplementary File 3, if sucrose is the biosynthetic product, values for *a*_*BS,M*_, *f*_*LET,BS*_ and *f*_*LET,M*_ can be derived from the Yin and Struik model and applied to *iTJC1414x4*, leading to a balanced energy budget, i.e., no ATP or NADPH surplus is detected.

### Constraining Photosynthetic Rates Based on Leaf Physiology

To set the photosynthetic assimilation rate in *iTJC1414x4*, we searched literature to define physiologically realistic values for daily photosynthetic rates of CO_2_ fixation for a sun-lit sorghum leaf under non-stressed field conditions. While direct measurements of total CO_2_ uptake have been reported for bioenergy grasses such as miscanthus and switchgrass (Dohleman et al., 2009), we could not find such data for sorghum. Reviewing literature for photosynthetic performance data specifically on *S. bicolor*, we found measurements of maximal photosynthetic CO_2_ fixation rates at mid-day. We therefore first derived an approximation to convert maximal photosynthetic CO_2_ fixation rates at mid-day to daily rates. Dohleman et al. (2009) measured leaf CO_2_ uptake rates for miscanthus and switchgrass for upper canopy sunlit leaves every 2 h on multiple days during the growing season. By plotting these uptake rates against time of day, the authors proposed a geometric method for estimating the daily photosynthesis rate. Here, we estimate the time course plot can be approximated with a parabolic function that peaks at mid-day and can be integrated to determine the daily rate. Accordingly, we defined the integral of daily CO_2_ fixation as 2/3 × (maximal photosynthetic CO_2_ fixation) × daylength (Supplementary Figure 3) and used this relation to estimate daily CO_2_ uptake rates for sorghum leaves based on mid-day photosynthetic rates. Around mid-day, under non-stressed conditions and at highest photosynthetic photon flux density, CO_2_ uptake rates measured for 22 *S. bicolor* genotypes ranged from 23.5 to 44.8 μmol CO_2_/m^2^/s, with an average of 38.2 μmol CO_2_/m^2^/s (Peng et al., 1991). In another study, under similar conditions, a range of 43.3–56.9 μmol CO_2_/m^2^/s was measured for 26 sorghum genotypes (Balota et al., 2008). Based on the two studies, we chose 40 μmol CO_2_/m^2^/s as a conservative estimate for the maximal photosynthetic rate. Using the parabola approximation with a daylength of 14 h, the CO_2_ uptake from the environment into the leaf during the daylight period is 1.344 mol CO_2_/m^2^/day.

In addition to the photosynthetic rates, it is important to consider energetic losses associated with cellular maintenance activities. This refers, for example, to energetic expenditures for maintenance of membrane gradients or continuous turnover of biopolymers. In plant FBA models such energetic costs are often classified as unspecified ATP as well as NADPH consumption (Sweetlove et al., 2013). To mimic energetic burdens of cellular maintenance, each cellular sub-model of *iTJC1414x4* includes a generic reaction to hydrolyze ATP (“*GenericATPasemod*”) and one to oxidize NADPH (“*GenericNADPOxidasemod*”). Based on the setup of other diel models of C_3_ and crassulacean acid metabolism (CAM) leaf metabolism (Cheung et al., 2014; Shaw and Cheung, 2018), we assumed here the maintenance costs to be the same in the light and dark phases, and we adopted a 3:1 ratio between ATP and NADPH drain fluxes (Cheung et al., 2013). To estimate dark respiration during the daytime we used a light response curve of sorghum leaves shown in Figure 4 of Wang et al. (2019). From this figure we determined the maximal photosynthetic rate for adaxial illuminated leaves was 38 μmol CO_2_/m^2^/s, which is close to the maximal photosynthetic rate of 40 μmol CO_2_/m^2^/s we considered here. The light response curve shows a linear increase in photosynthetic response up to a light intensity of about 500 μmol photon/m^2^/s with a slope of 0.064 mol CO_2_/mol photon and the dark respiration intersect of the linear was determined to be at −2.6 μmol CO_2_/m^2^/s. In another study evaluating five sorghum genotypes under field-grown conditions, respiration rates of dark-adapted leaves were on average 1.4 μmol CO_2_/m^2^/s (Li et al., 2021). We therefore consider the dark respiration intersect of 2.6 μmol CO_2_/m^2^/s to represent a rather conservative estimate. Assuming this value is constant over the 14 h daytime, the daily CO_2_ export rate is 0.131 mol CO_2_/m^2^/day. The model was adjusted to this dark respiration rate using the generic maintenance reactions (“*GenericATPasemod*,” “*GenericNADPOxidasemod*”), as detailed in Supplementary Figure 4.

### Aggregation of Network Fluxes for Quantitative Visualization

To give a summarizing overview of the flux solution space in *iTJC1414x4*, we defined a set of network projections that map selected groups of *iTJC1414x4* reactions onto a lumped reaction network as described before (Schwender and Hay, 2012). In short, a bilevel optimization as in Flux Variability Analysis is applied. However, the objective in the secondary optimizations is not to minimize/maximize a single reaction but a linear combination of reactions so that in effect the minimum and maximum bounds for the total flux between lumped metabolite pools is revealed. For example, to aggregate all conversions between oxaloacetate and malate by all malate dehydrogenase enzyme isoforms in the BSC-day sub-model, and taking into consideration the directionality of each isoform, the objective function is to maximize/minimize the following sum: “*bsDayMALATEDEHYDROGENASENADPRXNchloroplast* – *bsDayMalateDHPeroxisome* – *bsDayMalateDH* – *bsDayMALATEDEHRXNmitochondrion*.” All used summation terms of this type are listed in Supplementary Table 7.

### Energy Demands for Various Potential Products of Photosynthetic CO_2_ Assimilation

Independent of specific metabolic pathways, any oxygenic photosynthetic process converting CO_2_ and water into a carbon-reduced product and oxygen can be defined by its chemical and redox balance. Accordingly, we derived a general calculation scheme to derive the chemical balance of formation and the average oxidation state of carbon and applied it to various carbon-reduced compounds (Supplementary File 3). In addition to the chemical balances, we derived the overall balances of formation from CO_2_ and water by plant specific biochemical pathways. For this purpose, we defined a minimal stoichiometric model representing canonical pathways of the CBB cycle of C_3_ photosynthesis, the photorespiratory cycle, and sucrose, fatty acid, and TAG synthesis. This one-compartment model can only exchange sucrose, TAG, CO_2_, oxygen, and water with the environment. Using the software tool METATOOL (Schuster et al., 1999, 2000), the mass balances of all possible conversions of external metabolites into sucrose or TAG can be determined as Elemental Flux Modes (see Supplementary File 3 for details and Supplementary File 4 for METATOOL input files). Reaction coupling with dummy species “PSATP” and “PSH2” reveals the biosynthetic demands in ATP and reducing equivalents.

## Results

### Diel Model of NADP-Malic Enzyme C_4_ Metabolism in *Sorghum bicolor*

The sorghum model *iTJC1414x4* is comprised of four sub-models representing leaf MC and BSCs under day and night conditions (Figure 1). Each of the sub-models has over 1,000 reactions, 750 metabolites, and 7 intracellular compartments (Table 1). The day and night parts of the model are connected by a transitory starch pool. The fraction of assimilated carbon that is converted into (transitory) starch is consumed at the same rate in the dark model by biosynthetic or respiratory processes (Figure 1). Note that the simulated flux distributions are to be understood as integrals over the entire day. *iTJC1414x4* was constructed using the *Z. mays* metabolic reconstruction of leaf C_4_ metabolism *iEB2140x2* (Bogart and Myers, 2016) as a template. We added 380 reactions and modified over 100 reactions per sub-model, thereby correcting mostly for subcellular localization or cofactor specificity of fatty acid biosynthetic reactions. Effort at manual curation was particularly focused at lipid metabolism, which was done mostly in reference to plant-specific reactions as outlined in Li-Beisson et al. (2013). In short, chloroplast localized fatty acid biosynthesis was defined to produce palmitic acid (C16:0, a 16-carbon fatty acid with zero double bonds), stearic acid (C18:0) and oleic acid (C18:1), which are exported to the cytosol as free fatty acids (Li-Beisson et al., 2013). Cytosol/endoplasmic reticulum-localized lipid metabolism was set up as a sub-network specifying the biosynthesis of PC, DAG, and TAG with distinct molecular species that are composed of five acyl chain types, which are palmitate, stearate, oleate, and the polyunsaturated linoleic acid (C18:2) and linolenic acid (C18:3). The linoleate and linolenate species derive by desaturation reactions of acyl chains bound to PC (Li-Beisson et al., 2013). Degradation of TAG and fatty acids is given by TAG lipase reactions, by core activities of β-oxidation of the modeled fatty acid species (Li-Beisson et al., 2013) and by functions of the glyoxylate cycle (Graham, 2008). Besides lipid components, the model is able to synthesize protein, cellulose, hemicellulose, soluble sugars, and lignin (Supplementary Table 3).

**TABLE 1:** Basic statistics of *iTJC141414x4* sub-models.

**Reactions**	1006
Gene-associated	464
Metabolic	792
Transport	109
Sink	105
**Genes**	1,414
Differentially expressed	52
**Metabolites**	813
Cytosol	375
Plastid	211
Mitochondria	38
Peroxisome	155
Biomass Sink	60
Extracellular	28
**Compartments**	12
Intracellular	7
Inter- or extracellular	5

*Attribute counts are per sub-model in the iTJC1414x4 diel model when applied to mature Sorghum bicolor leaf cells. Sub-models correspond to cell type (bundle sheath or mesophyll) and timeframe (day or night). Reactions constrained to be inactive in one cell type due to differential gene expression are described in Supplementary Table 8.*

Of the 2,133 *Z. mays* genes associated with reactions in the *iEB2140* single-cell model, 85% of the genes had an *S. bicolor* ortholog and 92% of the reactions had *S. bicolor* orthologs for most of the associated maize genes (Supplementary Figures 1B,C). Together, these support the core *iEB2140x2* model being transferable from maize to sorghum. To integrate information on cell type specific metabolism, we analyzed differential gene expression data for *S. bicolor* MCs and BSCs, that was generated by Döring et al. (2016) using Illumina sequencing and SuperSage analysis. Of the 1,414 sorghum genes associated with reactions in *iTJC1414x4* (Table 1), 52 were found to be differentially expressed if either sequencing method was used. To be consistent with this data, we constrained nine MC and three BSC reactions to be inactive because all of their associated genes were found to be strongly preferentially expressed in the opposing cell (Supplementary Table 8).

### On a per Carbon Basis, Triacylglycerol Is About 30% More Energy Dense Than Sucrose

Prior to using *iTJC1414x4* to model TAG biosynthesis, we assessed energy content and biosynthetic costs of photo-assimilation independent from specific biochemical pathways. For a process of oxygenic photosynthesis that converts CO_2_ and water into a reduced product, the physicochemical limits are given by the chemical balance. Table 2 provides a list of reduced theoretical products, including organic acids, carbohydrates, lipids, and fossil fuel derived reduced carbon compounds, sorted by carbon reduction states and physicochemical demands in reducing equivalents. Five of the TAG molecular species listed are representative of the major fatty acids that are commonly accumulated in TAGs in crop plants, in particular palmitate, stearate, oleate, linoleate, and linolenate (Table 2). The amounts of required reducing equivalents and energy contents listed in Table 2 are derived from the elemental composition of each listed compound (for details see Supplementary File 3). Therefore, by averaging the listed TAG species, the properties of any molecular species composed of the mentioned five major fatty acids can be derived. On a per weight basis, the TAG species are on average 2.39-times more energy dense than sucrose (ratio ranging from 2.37 to 2.44; Table 2). However, since this study aims at analyzing partitioning of photo-assimilate into different reduced carbon compounds, it is important to also consider the molar quantity of assimilated CO_2_ (i.e., carbon) as a basis for comparing energy content and biosynthetic demands. If considered on a per carbon basis, sorghum wild-type leaf TAG only has 1.29-fold higher energy content than sucrose, while high-oil leaf TAG is 1.31-fold higher and different TAG molecular species are on average 1.3-fold higher in energy content than sucrose (ranging from 1.27 to 1.34; Table 2). As expected, because the biosynthetic process can be understood as the transfer of reducing equivalents onto carbon, the differences in per carbon energy content and per carbon reducing equivalents demands are similar.

**TABLE 2:** Energetic demands in reducing equivalents and energy density for theoretical products of photosynthesis.

Product	Formula	Average oxidation state of carbon	Required reducing equivalents per mol carbon	Energy density (kJ/mol carbon)^9^	Energy density (kJ/g)^9^
Citric acid	C_6_H_8_O_7_	1	1.50 (0.75)	−326.77 (0.69)	−10.21 (0.62)
Sucrose	C_12_H_22_O_11_	0	2.00	−470.28	−16.50
Glucose polymer (cellulose/starch)	C_6_H_10_O_5_	0	2.00 (1.00)	−452.83 (0.96)	−16.76 (1.02)
Lignin^1^	C_10.3_H_13.9_O_3.3_	−0.67	2.33 (1.17)	−515.48 (1.1)	−27.87 (1.69)
Trilinolenin^2^	C_57_H_92_O_6_	−1.40	2.70 (1.35)	−598.39 (1.27)	−39.05 (2.37)
Sorghum wild-type leaf TAG^3^	C_56.3_H_95_._0_O_6_	−1.48	2.74 (1.37)	−608.57 (1.29)	−39.47 (2.39)
Sorghum high-oil leaf TAG^4^	C_55.4_H_97.9_O_6_	−1.55	2.78 (1.39)	−616.90 (1.31)	−39.74 (2.41)
Trilinolein^5^	C_57_H_98_O_6_	−1.51	2.75 (1.38)	−606.24 (1.29)	−39.36 (2.39)
Triolein^6^	C_57_H_104_O_6_	−1.61	2.81 (1.40)	−615.78 (1.31)	−39.71 (2.41)
Tripalmitate^7^	C_51_H_98_O_6_	−1.69	2.84 (1.42)	−619.72 (1.32)	−39.15 (2.37)
Tristearate^8^	C_57_H_110_O_6_	−1.72	2.86 (1.43)	−628.19 (1.34)	−40.23 (2.44)
1-Dodecene	C_12_H_24_	−2	3.00 (1.50)	−660.49 (1.4)	−47.09 (2.85)
Ethanol	C_2_H_6_O	−2	3.00 (1.50)	−684 (1.45)	−29.71 (1.8)
Octane	C_8_H_18_	−2.25	3.13 (1.57)	−678.75 (1.44)	−47.63 (2.89)
Methane	CH_4_	−4	4.00 (2.00)	−890.00 (1.89)	−55.63 (3.37)

*In a photosynthesis process, photolysis of water leads to two-electron reducing equivalents used for reduction of CO_2_. The average carbon oxidation state and number of reducing equivalents needed for product synthesis were calculated based on the chemical and redox balances as described in Supplementary File 3. As a context, the energy densities of these potential biofuels are given along with those of traditional fuels octane, ethanol, and methane, the organic compound with the highest reduction state of carbon. Values in parentheses are ratios relative to sucrose.*

*^1^Polymer composition used in iTJC1414x4 (19% coumaryl alcohol, 29% coniferyl alcohol, and 52% sinapyl alcohol).*

*^2^1,2,3-Tri-(octadecatrienoyl)glycerol.*

*^3^Fatty acid composition measured by Vanhercke et al. (2019a) and used in iTJC1414x4 (13% C16:0, 4% C18:0, 3% C18:1, 21% C18:2, 60% C18:3).*

*^4^Fatty acid composition measured by Vanhercke et al. (2019a) (26% C16:0, 4% C18:0, 11% C18:1, 38% C18:2, 21% C18:3).*

*^5^1,2,3-Tri-(octadecadienoyl)glycerol.*

*^6^1,2,3-Tri-(octadecenoyl)glycerol.*

*^7^1,2,3-Tri-(hexadecanoyl)glycerol.*

*^8^1,2,3-Tri-(octadecanoyl)glycerol.*

*^9^Empirical values for enthalpy of combustion (ΔcH°) were taken from http://webbook.nist.gov/ or calculated as described in Schmidt-Rohr (2015).*

In addition, per CO_2_ incorporated, sorghum wild-type TAG synthesis requires 1.37-fold the amount of reducing equivalents as sucrose (values for other TAG compositions range from 1.35 to 1.43; Table 2). Since the demands for reducing equivalents listed in Table 2 derive from chemical balances, they represent a chemical minimum of energetic investments. As detailed in Supplementary File 3, the overall biosynthetic demands in reducing equivalents will raise above this minimum if metabolic pathways contain steps where electrons are transferred from pathway intermediates back to oxygen. For synthesis of TAG with sorghum fatty acid composition, the chemical minimum demand is 2.74 reducing equivalents per carbon (Table 2), while biosynthetic pathways demand 2.98 reducing equivalents. As detailed in Supplementary File 3, the difference is accounted for by the transfer of electrons to oxygen at the fatty acid desaturase steps. Relative to the total biosynthetic demands, 8.1% of reducing equivalents are transferred back to oxygen.

### Imbalance in Photosynthetic ATP and NADPH Supply and Demand

To analyze possible photo-assimilatory pathways for sucrose and TAG synthesis, we first used the single-cell model *iTJC1414* to simulate three scenarios of sucrose or TAG formation under limiting light conditions (Supplementary File 1). Table 3 shows the simulations in the case of C_3_ photosynthesis without photorespiration being active (see Supplementary Table 9 for consideration of photorespiration). In each case the overall demands in CO_2_ and H_2_O as well as O_2_ production are shown in Table 3 and are as expected by the chemical balances (see Supplementary File 3). For each scenario, the optimal flux solution space was further characterized by Flux Variability Analysis with subsequent classification of flux bounds into flux variability types as described before (Hay and Schwender, 2011). For optimal sucrose production, 48 reactions must be active (Table 3, scenario 1, “essential reactions”), while, in between alternative optimal flux states, 25 reactions can have zero or non-zero flux values (“non-essential reactions”). For TAG synthesis, a much larger number of reactions are non-essential than are essential (Table 3, scenario 2). While this might not be unexpected due to the complexity of the TAG biosynthetic network, inspection of the 618 non-essential reactions in scenario 2 revealed that the optimum TAG solution space allows many reactions to be active that should not be required for photo-assimilatory TAG synthesis (Supplementary Table 10). For example, invertase (“*RXN1461*”) was active in what appears to constitute a cycle of synthesis and degradation of sucrose. The Oxidative Pentose Phosphate Pathway (OPPP) can be active (e.g., “*GLU6PDEHYDROGRXNChloroplast*”) although in the photoautotrophic context, activity of OPPP and CBB cycle constitute a futile cycle with net ATP consumption (Sharkey and Weise, 2016). Altogether, the optimum solution space seems to feature numerous alternative solutions with ATP consuming futile cycles across the network that dissipate an apparent over-supply in ATP. To confirm this suspicion, we showed that this ATP consuming activity can be aggregated by maximizing a generic ATP consuming reaction, which we added to the network (“*ATPsurplus*”). If fixed to its maximal possible value under optimality, subsequent Flux Variability Analysis resulted in a much smaller solution space in terms of number of active reactions and futile cycles were no longer detectable (Table 3, scenario 3). In addition to the observations made in Table 3, simulations that include photorespiratory activity led to essentially the same conclusions on the occurrence of ATP surplus for TAG synthesis (Supplementary Table 9). The explanation for an over-supply (“surplus”) in ATP is found in the rigidity of the photosynthetic light-driven production of ATP and NADPH. As we will explore in more detail below, the lowest possible ratio at which ATP and NADPH can be supplied by the photosynthetic apparatus is at ATP/NADPH = 1.5. If the overall metabolic demands ratio for a particular biosynthetic product is below 1.5, stoichiometrically feasible optimal flux solutions will require the dissipation of surplus ATP. This model finding has the equivalence of an ATP/NADPH imbalance in the real process. The photosynthetic apparatus can be considered to deliver ATP and NADPH at fixed proportions, which should precisely match the proportions of the metabolic demands (Kramer and Evans, 2011). Chloroplasts are said to have limited capacity to adapt to variation in the relative demands in ATP and NADPH and a misalignment can stall the overall process (Kramer and Evans, 2011).

**TABLE 3:** Photo-assimilation of CO_2_ into sucrose or TAG using the single-cell model *iTJC1414*.

	Scenario (product)		
	1 (sucrose)	2 (TAG)	3 (TAG)
*ATPsurplus* flux constraint (*mol* / *mol* CO_2_)	0	0	0.468
**Quantum yield, mass balance**			
*Quantum yield* (*mol* CO_2_ / *mol* photon absorbed)	0.107	0.073	0.073
CO_2_ consumption (*mol* / *mol* product)	12	56.2	56.2
H_2_O consumption (*mol* / *mol* product)	11	47.4	47.4
O_2_ production (*mol* / *mol* product)	12	76.9	76.9
**Flux variability type characterization of optimum flux space**			
Essential reactions (all possible flux values ≠ 0)^1^	48	70	83
Non-essential reactions (flux values can be = 0)^2^	25	618	155
Reactions never used^3^	946	331	781

*Flux scenarios are in dependence of a fixed value for a generic ATP consuming reaction “ATPsurplus.” The optimum flux solution space was predicted by Flux Variability Analysis for maximal product flux, given limiting light and CO_2_ uptakes. The predicted balance in CO_2_, H_2_O, and O_2_ is listed and agrees with the chemical balances (see Supplementary File 3). In scenario 3 the flux variability solution space is characterized again after ATPsurplus was fixed to its maximum value under scenario 2. For each scenario, the optimal solution space was characterized according to flux variability types (Hay and Schwender, 2011): For all 1,018 reactions in iTJC1414, the range of individual flux values was categorized as indicated below (numerical tolerance 10^–6^). Values ± 1,000 are the upper/lower flux bounds used in the model. See Supplementary Table 10 for flux variability analysis output and flux variability categorizations. For the shown scenarios, photorespiration is inactive. For the same calculations under consideration of photorespiration, see Supplementary Table 9.*

*^1^“+,” “−,” “[+ +],” “[−−],” “[+ +1000],” “[−1000 −].”*

*^2^“[− +],” “[− +1000],” “[− 0],” “[0 +],” “[0 +1000],” “[−1000 +],” “[−1000 +1000],” “[−1000 0].”*

*^3^“0.”*

### Pathways for Photo-Assimilation of CO_2_ Into Sucrose and Triacylglycerol

**FIGURE 2:**
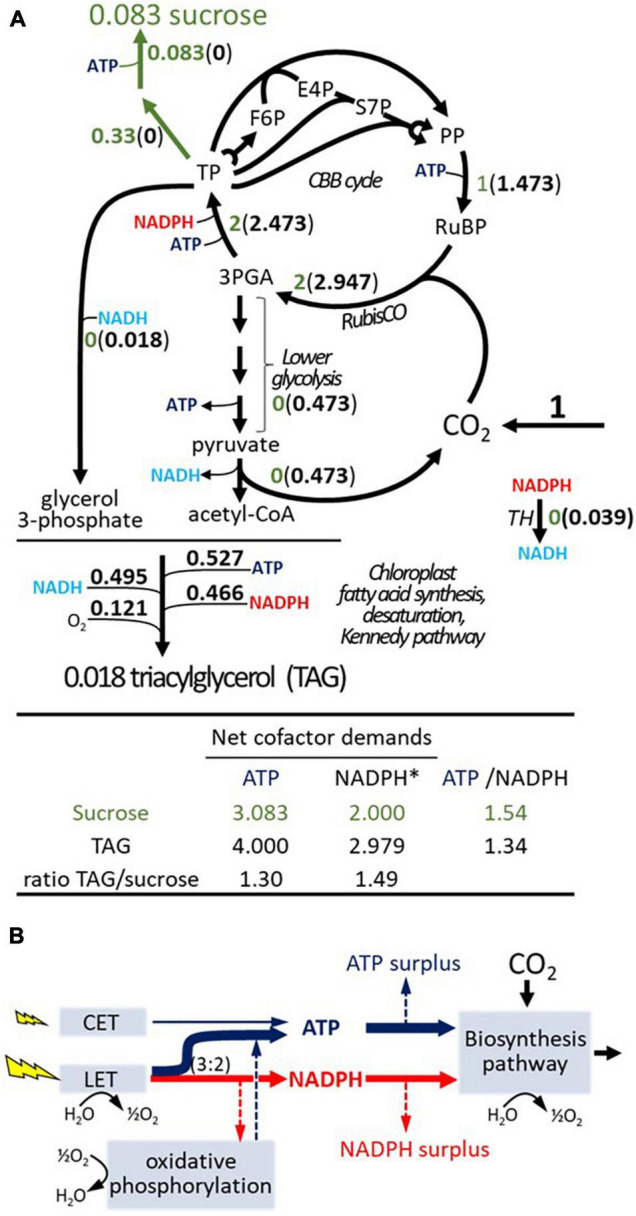
Basic stoichiometries for sucrose and TAG synthesis and cellular energy cofactor budget. **(A)** Stoichiometries for the assimilation of CO_2_ *via* the Calvin–Benson–Bassham (CBB) cycle into sucrose (green shading) or TAG based on balancing a generic plant stoichiometric model (see Supplementary File 3) with fatty acid composition of TAG as in *iTJC1414x4*. Flux values are normalized relative to one mol of CO_2_ net assimilation. Oxygen consumption shown for TAG synthesis is due to fatty acid desaturase reactions. **(B)** Balance of ATP and NADPH between supply (photosynthetic light reactions) and demands (biosynthetic pathway). In modeling scenarios, the light reactions are thought to supply ATP and NADPH at a fixed ratio. If the supply ratio is above the demand ratio, then a surplus in ATP supply occurs that must dissipate in other processes. If the supply ratio is lower than the demand ratio of a biosynthesis pathway, then an NADPH surplus should result. However, mitochondrial oxidative phosphorylation allows for adjustment of the supply ratio. Otherwise, a NADPH surplus exists. CET, cyclic electron transport; F6P, fructose 6-phosphate; GAP, glyceraldehyde 3-phosphate; LET, linear electron transport; 3-PGA, 3-phosphoglycerate; PP, pentose phosphates; RuBP, ribulose 1,5-bis-phosphate; RubisCO, ribulose 1,5-bis-phosphate carboxylase/oxygenase; S7P, sedoheptulose 7-phosphate; TH, unspecified transhydrogenase activity; TP, triose phosphate (e.g., glyceraldehyde 3-phosphate, dihydroxyacetone phosphate); TAG, triacylglycerol. *photosynthetic reducing equivalents.

To further explain the results on an ATP/NADPH imbalance, we assessed the metabolic demand independent of supply (Figure 2A). We defined a minimal stoichiometric model for CO_2_ assimilation, consisting of 44 reactions representing plant canonical pathways of the CBB cycle of C_3_ photosynthesis (Sharkey and Weise, 2012), the photorespiratory cycle (Peterhansel et al., 2010), sucrose synthesis (Stitt et al., 1987), the generation of acetyl-CoA directly from 3-PGA (Joyard et al., 2010), and a net equation for chloroplast fatty acid synthesis. To obtain the latter, the net balance from acetyl-CoA and energy cofactors was derived according to generic plant pathways (Li-Beisson et al., 2013) by assembling the stoichiometries of 47 reaction steps. Moreover, the minimal model is a subset of *iTJC1414* (see Supplementary File 3 for details). Applying Elementary Flux Modes analysis to the minimal model, the net balance for the conversion of CO_2_ and water to oxygen and sucrose or TAG can be obtained along with the energy cofactor requirements. Distinct flux modes were obtained for sucrose and TAG production; in each case one with the photorespiratory pathway being active and one without (Supplementary File 3). Figure 2A summarizes the resulting scenarios for sucrose and TAG synthesis if photorespiration is not active. Flux rates and energy cofactor demands are shown relative to the net assimilation of one *mol* of CO_2_. It is noticeable that, relative to sucrose production, CBB cycle fluxes are expected to be substantially higher if TAG is the product (Figure 2A). This is because, at the PDHp step, one-third of the carbon that is processed from 3-PGA toward fatty acid biosynthesis is released as CO_2_ and then re-fixed by RubisCO. Per CO_2_ assimilated into TAG, ATP-, and NADPH-demands are higher by 1.3- and 1.49-fold, respectively (Figure 2A). Similar increases are observed if photorespiration is active (see Supplementary File 3).

Considering ATP and NADPH relative to each other, the ATP/NADPH demand ratio for TAG synthesis is lower than that for sucrose (Figure 2A). The basic concept of an energy cofactor budget is outlined by the supply/demand scheme shown in Figure 2B. If the ATP/NADPH supply ratio is higher than the demand ratio for a biosynthetic product, then there remains an ATP surplus which must dissipate in a process other than biosynthesis. If the ATP/NADPH supply ratio is lower than the demand ratio, then there will be a surplus in reducing equivalents, which can be dissipated by activity of oxidative phosphorylation or by another oxidative process (Figure 2B). Based on the stoichiometric setup of light reactions in *iTJC1414*, the ATP/NADPH ratio supplied by the LET chain is 1.5 (Supplementary File 3). Note that there are some uncertainties about the true ratio of ATP/NADPH output from LET found in plants, but most literature sources determine the ratio as being close to 1.5 (Noctor and Foyer, 1998; Allen, 2003; Kramer and Evans, 2011). Thus, considering LET to be active with only a very small additional ATP contribution by CET, the photosynthetic supply is sufficient to match the demands of 1.54 ATP per NADPH for sucrose synthesis (Figure 2B). Supposing that the ATP and NADPH supply is tailored this way for sucrose synthesis with a rigid supply ratio of 1.54, the demand ratio of 1.34 for TAG (Figure 2A) will cause an ATP surplus. If NADPH supply and demand are aligned, the ATP surplus amounts to 13% of the production [(1.54−1.34)/1.54]. Based on the principle outlined in Figure 2B we modeled and assessed the effects of a rigid energy cofactor supply for the two cell types of C_4_ metabolism in *iTJC1414x4*. This allowed to define a reference scenario for CO_2_ assimilation into sucrose, where photosynthetic ATP and NADPH production are in balance with metabolic demands in both cell types. Based on this reference state, the energy balance in TAG biosynthesis scenarios can be evaluated.

### Obtaining a Balanced Energy Budget in C_4_ Photosynthesis

In C_4_ photosynthesis, different metabolic activities must be coordinated across the two cell types. Before applying sorghum-specific constraints to the distribution of light energy among the two cell types in *iTJC1414x4*, we first verified the basic configuration where the light uptake fluxes and the relative activities of LET and CET were freely adjustable. If sucrose synthesis was simulated by minimization of total light uptake, it was found that with the overall photosynthetic energy cofactor supply is 3.647 ATP and 2.431 NADPH per mol CO_2_ assimilated (Table 4, scenario 1, sucrose). Since sucrose synthesis requires 2 NADPH per CO_2_ assimilated (Table 2), there is an oversupply of 0.431 NADPH. Further inspection of the flux scenario revealed that this amount of reducing equivalents is consumed by mitochondrial oxidative phosphorylation, which generates additional ATP, resulting in an adjusted overall cofactor supply of 5.083 ATP and 2 NADPH per mol CO_2_ (Table 4, scenario 1, sucrose). This adjusted supply meets the above established general expectation for the basic photosynthetic demands of 3.083 ATP and 2 NADPH per mol CO_2_ (Figure 2A), after it is also considered that the operation of the C_4_ cycle incurs an additional 2 ATP/CO_2_ at the pyruvate-phosphate dikinase (PPDK) step (Figure 1). Potentially, the additional ATP could be generated by CET. However, the basic sucrose synthesis flux scenario predicts CET to be inactive in both cell types (Supplementary Table 7). Flux modes analysis of the isolated ATP and NADPH generating system shows that ATP production *via* combined LET and oxidative phosphorylation activity has higher quantum use efficiency than ATP production *via* CET (Supplementary File 3).

**TABLE 4:** Energy budgets for photo-assimilation of CO_2_ in *iTJC1414x4*.

Model scenario products	Photosynthetic cofactor supply across the BSC and MC (*mol* / *mol* CO_2_ fixed)				Adjusted cofactor supply across BS- and M cells (*mol* / *mol* CO_2_ fixed)			Φ_*CO2*_ (*mol* CO_2_ / *mol* photon)
	ATP	NADPH	Δ ATP^1^	OP^2^	ATP	NADPH	ATP/NADPH	
**1. Constraints: free adjustable light uptake fluxes (i.e., no restrictions on *a*_*BS,M*_, *f*_*LET,BS*_, *f*_*LET,M*_)**								
Sucrose	3.647	2.431	0.000	0.431	5.083	2.000	2.54	0.089
TAG (BSC)	4.797	3.198	0.000	0.219	5.527	2.979	1.86	0.068
TAG (MC)	5.090	3.394	0.000	0.415	6.473	2.979	2.17	0.064
Ratio TAG (BSC)/sucrose	1.32	1.32			1.09	1.49		0.76
Ratio TAG (MC)/sucrose	1.40	1.40			1.27	1.49		0.72

**2. Constraints: *a*_*BS,M*_ = 0.389, *f*_*LET,BS*_ = 0.032, *f*_*LET,M*_ = 0.933**								
Sucrose^3^	5.083	2.000	0.000	0.000	5.083	2.000	2.54	0.073
TAG (BSC)	7.571	2.979	2.044	0.000	5.527	2.979	1.86	0.049
TAG (MC)	7.571	2.979	1.097	0.000	6.473	2.979	2.17	0.049
Ratio TAG (BSC)/sucrose	1.49	1.49			1.09	1.49		0.67
Ratio TAG (MC)/sucrose	1.49	1.49			1.27	1.49		0.67

**3. Constraints: *a*_*BS,M*_ = 0.389, *f*_*LET,BS*_ = 0.032, *f*_*LET,M*_ = 0.895, leaf physiological constraints**								
Assimilate export^4^	6.236	2.330	0.000	0.000	6.236	2.330	2.68	0.060
Oil droplets (BSC)^4,5^	8.573	3.203	1.487	0.000	7.086	3.203	2.21	0.044
Oil droplets (MC)^4^	8.573	3.203	0.729	0.000	7.844	3.203	2.45	0.044
Ratio Oil droplets (BSC)/assimilate	1.37	1.37			1.14	1.37		0.73
Ratio Oil droplets (MC)/assimilate	1.37	1.37			1.26	1.37		0.73

*Flux scenarios are based on first minimizing the light uptake fluxes for CO_2_ assimilation into sucrose at a fixed rate, subject to the constraints listed for each scenario. Then, given the obtained light flux limits, flux states are generated with maximum possible TAG biosynthesis rate in the BSC or MC. Detailed results are listed in Supplementary Table 7. Shown are supply of ATP and NADPH, directly from photosynthetic light reactions and after adjustments that account for ATP surplus (ΔATP) and the activity of oxidative phosphorylation (OP) in mitochondria (see Supplementary File 3). The adjusted energy budget equals the cellular metabolic demands plus cellular maintenance costs. For scenarios 1 and 2, leaf physiological constraints, including cellular maintenance, CO_2_ leakage and photorespiration (Supplementary Table 2), were omitted to obtain the energy cofactor balances solely based on photo assimilation of CO_2_ into sucrose or TAG.*

*^1^ATP surplus encompassed in the photosynthetic ATP value (see Figure 2B and section “Materials and Methods”).*

*^2^Amount of reducing equivalents (mol / mol CO_2_ fixed) primarily produced by photosynthesis that was consumed in mitochondrial oxidative phosphorylation to adjust supplies (1 NADPH yields 3.33 ATP).*

**FIGURE 3:**
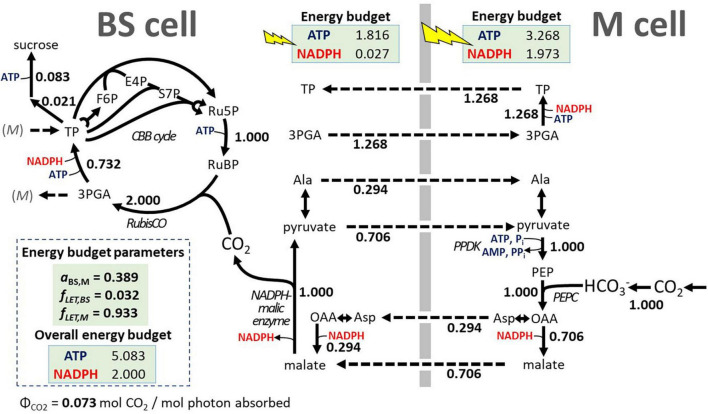
Effect of energy budget parameters on the C_4_ photosynthesis scheme in *iTJC1414x4*. Flux values are shown relative to uptake of one unit CO_2_. The shown sorghum-specific parameters for light flux distribution (energy budget parameters) were derived from a cell-type-specific electron transport model for C_4_ plants by Yin and Struik (2018) and cause BSC- and MC-specific production of ATP and NADPH by photosynthetic light reactions to balance the photosynthetic supplies and metabolic demands. Note that at the PPDK step, AMP needs to be recycled, leading to a net demand of 2 mol ATP for PEP regeneration. Flux values are obtained from the primary FBA solution using flux projections (see section “Materials and Methods” and Supplementary Table 7, model scenario 3). 3PGA, 3-phosphoglycerate; *a*_*BS,M*_, BS:M ratio in light absorption; Ala, alanine; Asp, aspartate; BS cell, bundle sheath cell; CBB, Calvin–Benson–Bassham; E4P, D-erythrose-4-phosphate; F6P, D-fructose-6-phosphate; *f*_*LET,BS*_, fraction of BS cell absorbed irradiance that is used for linear electron transport; *f*_*LET,M*_, fraction of M cell absorbed irradiance that is used for linear electron transport; M cell, mesophyll cell; OAA, oxaloacetate; PEP, phosphoenolpyruvate; PPDK, pyruvate phosphate dikinase; Ru5P, D-ribose-5-phosphate; RubisCO, ribulose 1,5 bisphosphate carboxylase oxygenase; RuBP, D-ribulose-1,5-bisphosphate; S7P, D-sedoheptulose-7-phosphate; TP, triose phosphates; ΦCO_2_, absorbed quantum yield for CO_2_ uptake (mol CO_2_ / mol photon).

*^3^Scenario of Figure 3.*

*^4^In these scenarios, phloem-exported photo-assimilates (sucrose and amino acids, Supplementary Table 4) and oil droplets (TAG, and oleosin, Supplementary Table 5) are the assimilatory products. In addition, 17% of net CO_2_ is fixed into transitory starch that feeds respiratory activity on the night-side.*

**FIGURE 4:**
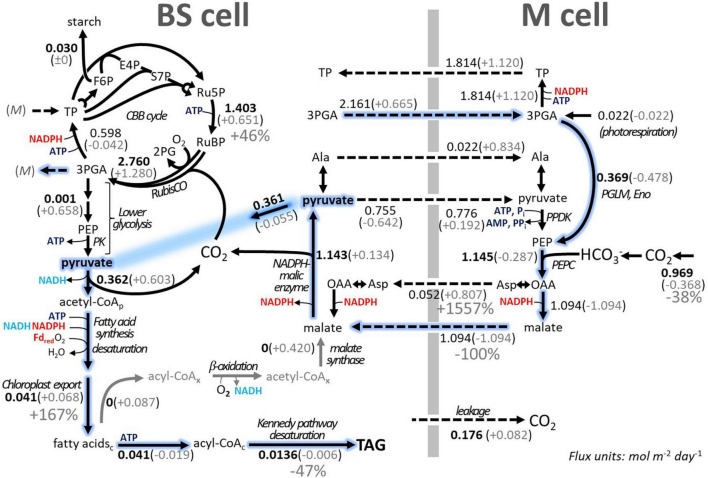
Schematic overview of TAG biosynthesis in the BSC of *iTJC1414x4* and impact of fatty acid futile cycling on TAG yield. An uncompartmentalized schematic view of the day sub-models is given summarizing major fluxes, which were predicted based on multilevel optimization (flux projections, see section “Materials and Methods”). Bolded flux values are invariable in the FBA solution space. Flux units are mol m^–2^ day^–1^ and complete flux solutions are listed in Supplementary Table 7. Gray values given in parentheses are changes in flux values upon enforcing fivefold cycling [i.e., fatty acid biosynthesis / (3 × TAG storage flux) = 5]. Gray arrows summarize the degradation of fatty acids and formation of malate, a process that extends across the cytosol, mitochondrial, and peroxisomal compartments. Dashed arrows indicate metabolite transport through plasmodesmata between cell types. The BSC pyruvate pool is shown twice as emphasized in blue. Also marked in blue is a scheme where generation of pyruvate destined to fatty acid synthesis intersects with the 3PGA/triose phosphate shuttle and the C_4_ shuttle. Subscripts “c,” “p,” and “x” indicate metabolites specific to the cytosolic, plastidic, and peroxisomal compartment, respectively. 3-PGA, 3-phosphoglycerate; Ala, alanine; CBB, Calvin–Benson–Bassham; Eno, enolase; F6P, fructose 6-phosphate; Fdred, reduced ferredoxin; OAA, oxaloacetate; PEPC, phoephoenolpyruvate carboxylase; PGLM, phosphoglyceromutase; Pi, inorganic phosphate; PK, pyruvate kinase; PPDK, pyruvate, phosphate dikinase; PPi, inorganic pyrophosphate; Ru5P, ribulose 5-phosphate; RubisCO, ribulose 1,5 bisphosphate carboxylase oxygenase; RuBP, ribulose 1,5-bis-phosphate; S7P, sedoheptulose 7-phosphate; TAG, triacylglycerol; TP, triose phosphate.

*^5^Scenario of Figure 4.*

To develop a sorghum-specific configuration of light energy distribution, we assumed that a typical mature sorghum leaf must have a photosynthetic energy cofactor supply that is well balanced with the metabolic demands of sucrose synthesis. To define a typical sorghum light distribution pattern with energy balance for sucrose synthesis, we relied on a model of cell-type specific electron transport in C_4_ photosynthesis by Yin and Struik (2018). The analytical model predicts the energy budgets for the major sub-types of C_4_ photosynthesis based on the cofactor demands of the CBB cycle, taking sub-type specific variations in the C_4_ cycles into consideration and by estimating photosynthetic production capacity based on leaf morphology and the distribution of chlorophyll and photosystems I and II between BSCs and MCs. We used the Yin and Struik (2018) model to predict three parameters to be incorporated into our sorghum FBA model: the ratio of light absorptance between the BSC and MC (*a*_*BS,M*_) and, for the BSC and MC, respectively, the fraction of absorbed light that drives LET (*f*_*LETBS*_, *f*_*LETM*_). With sorghum specific parameters and defining sucrose synthesis as the energy consuming metabolic activity, the Yin and Struik (2018) model predicts a balanced C_4_ energy budget at *a*_*BS,M*_ = 0.398, *f*_*LETBS*_ = 0.032, and *f*_*LETM*_ = 0.933 (see Supplementary File 3 for details). After the three energy budget parameters were applied to *iTJC1414x4* (Equations 6, 7), no ATP surplus or oxidative phosphorylation adjustment was detected for the two cell types (Table 4, scenario 2, sucrose). If, instead of sucrose, TAG is the product in the BSC or the MC, then an ATP surplus of 2.044 or 1.097 ATP per CO_2_ fixed can be detected, respectively (Table 4, scenario 2, TAG). This means that using these energy budget parameters, a state was defined in *iTJC1414x4* where cell-type specific photosynthetic ATP and NADPH supplies are in balance with cell-type specific demands for sucrose synthesis. Figure 3 illustrates the functioning of C_4_ metabolism under this condition. With the 3-PGA/triose phosphate shuttle operating, 37% of the reduction of 3-PGA to GAP takes place in MC, meaning that 37% of the ATP and NADPH demand at this step in the CBB cycle is assigned to the MC. The malate/pyruvate shuttle mechanism, which transfers both CO_2_ and reducing equivalents, is dominant. However, 29.4% of the CO_2_ is transported *via* the aspartate/alanine mechanism, which transports CO_2_ only (Figure 3). This means that only 70.6% of the possible capacity to move NADPH with the C_4_ shuttle is used. Overall, the imposition of the energy budget parameters appears to create a realistic reference state with balanced energy budget for sucrose synthesis.

### Definition of a Physiologically Relevant Mature Leaf Reference State With Balanced Energy Budget in C_4_ Photosynthesis

In Figure 3 we consider sucrose biosynthetic costs only. To simulate carbon assimilation more realistically, we need to calibrate the model to the overall photosynthetic capacity of a sorghum leaf and incorporate estimates on additional energetic costs due to photorespiration, CO_2_ leaking back from BSC to MC and costs related to respiratory activity. As described in section “Materials and Methods,” to set the photosynthetic capacity we derived a typical daily CO_2_ assimilation rate based on a survey of literature-reported maximal photosynthetic CO_2_ fixation rates at mid-day, daily time course integrals as well as measured leaf dark respiration rates Accordingly, we estimate the total daily CO_2_ assimilation rate for sucrose synthesis in a mature sorghum leaf to be 1.344 mol CO_2_/m^2^/day, with a respiratory loss of 0.131 mol CO_2_/m^2^/day. We next used these two rates in an iterative cycle that is outlined in Supplementary Figure 4 to set the model’s light uptake and maintenance fluxes as well as to adjust the energy budget parameters, beginning with the values for *a*_*BS,M*_, *f*_*LET,BS*_, and *f*_*LET,M*_ obtained above from the Yin and Struik model (Table 4, scenario 2).

In short, we first determined the minimal light levels for which the combined net daily CO_2_ assimilation and respiratory loss (1.475 mol CO_2_/m^2^/day) can be converted into sucrose. Second, while keeping these light fluxes constant, we raised the day-time maintenance cost so that the combined CO_2_ rate was reduced back to the net daily CO_2_ assimilation rate of 1.344 mol CO_2_/m^2^/day. Assuming that maintenance respiratory loss during the 14 h day-time also applies to the night-time (10 h), the night-time ATP and NADPH drain reactions were set to 10/14 of the daytime rates. Third, to obtain a mature leaf reference state, the photo-assimilate export rate was maximized. The iterative cycle was repeated by adjusting *f*_*LET,M*_ until, across the two cell types, no ATP surplus or supplemental ATP production by mitochondrial oxidative phosphorylation could be detected. This means that at the final state the photosynthetic production of ATP and NADPH was balanced with the overall demands for synthesis of the exported photo-assimilate. In result, we obtained light uptake fluxes of 6.30 and 15.82 mol photons/m^2^/day for the BSC and MC, respectively. The daytime ATP and NADPH drain reactions were determined to be 0.369 and 0.123 mol/m^2^/day, respectively. The corresponding nighttime values for ATP and NADPH drain reactions were 0.264 and 0.088 mol/m^2^/day, respectively. These settings for nighttime maintenance cause a nighttime respiratory CO_2_ loss that is 13.4% of the CO_2_ uptake flux. Nighttime respiratory loss of non-stressed *S. bicolor* plants has been estimated before to be between 10 and 16% of the daily photosynthesis rate (Hodges et al., 1979). We therefore consider the respiratory loss of 13.4% to be realistic. The balanced C_4_ energy budget was obtained after an adjustment of the value for *f*_*LET,M*_ from 0.933 (Table 4, scenario 2, sucrose) to 0.895, while *a*_*BS,M*_ and *f*_*LET,BS*_ were kept at their formerly determined values (Table 4, scenario 3, assimilate export). The predicted absorbed quantum yield for CO_2_ uptake is 0.060 mol CO_2_ / mol photons (Table 4, scenario 3, assimilate export), which is similar to the measured value for sorghum leaves of 0.061 mol CO_2_ / mol photons (Ehleringer and Pearcy, 1983). Finally, this scenario was taken to define a mature leaf reference state with balanced energy budget based on which carbon partitioning between assimilate export and oil droplet deposition can be further characterized. All constraints that define this state are listed in Supplementary Table 2 and Figure 1 highlights the constraints related to light flux, photorespiration, CO_2_ leakage and maintenance cost.

### Triacylglycerol Synthesis Causes ATP Surplus

By default, TAG biosynthesis and accumulation can take place in all four sub-models of *iTJC1414x4*. Given the above reference state, we tested the efficiency of conversion of CO_2_ into TAG in all four sub-models (Supplementary Figure 5). Overall, TAG production can take place in the BSC- and MC-day models at the same rate. In the dark models maximal TAG production is reduced by less than 1%, relative to the maximal day rate (Supplementary Figure 5). If energy budget parameters are applied (Table 4), the photosynthetic energy budgets are balanced for sucrose synthesis, while for TAG synthesis, a substantial ATP surplus is detected. For scenario 2 (Table 4), the ATP surplus values of 2.044 and 1.097 for TAG production in the BSC and MC, respectively, amount to 27 and 14.5% of the photosynthetic ATP production. For scenario 3 (Table 4) these percentages are 17.3 and 8.5%, respectively. ATP surplus under TAG synthesis does not occur when light flux distribution is freely adjustable (Table 4, scenario 1), which demonstrates that the ATP surplus is a consequence of application of the energy budget parameters. The model prediction of ATP surplus suggests that, unless there are mechanisms to flexibly adjust light energy input or to discharge the surplus in ATP in another process, high rates of TAG synthesis in a leaf might be stalled due to energy cofactor imbalances. To further explore model predictions of TAG biosynthesis, a scenario of daytime TAG accumulation in BSCs is further detailed in Figure 4. As seen for sucrose synthesis (Figure 3), CO_2_ from the environment is moved from MC to BSC *via* the malate/pyruvate shuttle mechanism with minor participation of the aspartate/alanine shuttle. In contrast to the sucrose synthesis scheme, a part of the pyruvate produced by NADP-ME in the BSC serves as a precursor to acetyl-CoA and fatty acid biosynthesis (Figure 4). The same amount of pyruvate that is removed from the pyruvate/malate shuttle cycle is replenished in MCs by conversion of 3-PGA to phosphoenolpyruvate. Altogether, a scheme emerges by which pyruvate destined to fatty acid synthesis is generated from 3-PGA *via* phosphoenolpyruvate under participation of the 3-PGA/triose phosphate shuttle and the C_4_ shuttles, while omitting the pyruvate kinase and PPDK steps (Figure 4, blue trace). The scheme is cost-saving relative to direct conversion of 3-PGA to pyruvate in BSC, since generating phosphoenolpyruvate in the C_4_ cycle with PPDK comes at a net cost of 2 ATP. However, the overall savings per unit pyruvate entering fatty acid synthesis is only one ATP since using pyruvate kinase in the BSC would gain one ATP (Figure 4).

### Shifts in Energetic Demands Associated With a Trade-off of Assimilate Export vs. Triacylglycerol Biosynthesis

**FIGURE 5:**
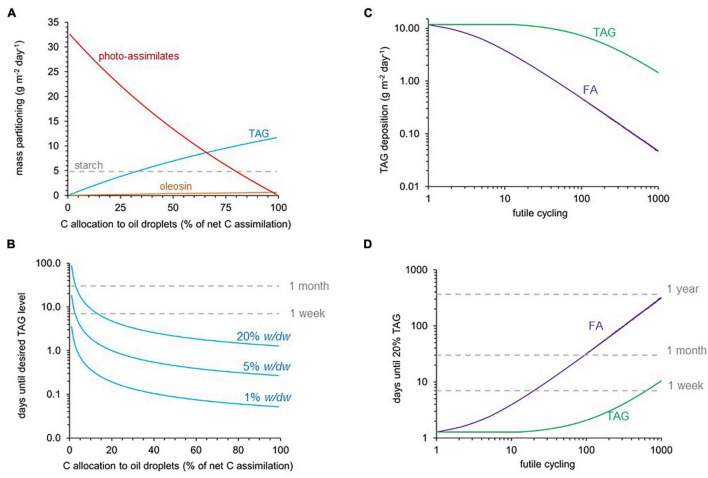
Model simulations for TAG accumulation in mature leaves. Daily photo-assimilate export (sucrose and amino acids) and TAG accumulation was modeled for mature *Sorghum bicolor* leaves under full-sun exposure (*iTJC1414x4*). See Supplementary Table 2 for main model constraints. **(A)** Mass partitioning of primary photo-assimilates between photo-assimilates (exported), starch (transitory), and TAG, which is deposited in the leaf with oleosin. Starch is not counted as net assimilation since it is completely degraded by night respiration activities. **(B)** Time period until a target level (1, 5, 20% w/dw) of TAG accumulation is reached, in dependence of the fraction of assimilated carbon being allocated to oil droplets (i.e., TAG and oleosin synthesis). The effects of TAG (purple) and FA (green) futile cycles on **(C)** TAG deposition and **(D)** the time needed to reach 20% (w/dw) TAG in leaf tissue if 100% of net photo-assimilated carbon is partitioned into oil droplets. TAG or fatty acids are synthesized up to 1,000-fold in excess over what is stored as TAG in oil droplets. Cycling units are mol TAG synthesis per mol TAG accumulated in oil droplets (TAG cycling) and mol fatty acid synthesis per mol fatty acid stored in oil droplets (FA cycling) (see section “Materials and Methods,” Equations 4, 5).

The amount of TAG that is accumulated in a leaf will depend on the fraction of photo-assimilate that can be re-directed into oil droplet biosynthesis by a metabolic engineering approach and by the number of days the accumulation can take place before the material is harvested. Imposition of a range of carbon partitioning ratios is demonstrated in Figures 5A,B. Carbon allocation is shifted between photo-assimilate export and oil droplet deposition while starch is synthesized at a constant rate of 4.8 g/m^2^/day (Figure 5A), which reflects a constant loss of daily net carbon assimilation by nighttime respiration. As carbon allocation toward oil droplets increased from 0 to 100% at the expense of photo-assimilate synthesis, the TAG mass flux as well as the carbon molar flux into TAG deposition did not increase linearly, but with a slightly downward bending trend (Figure 5). This indicates that net carbon fixation decreases as TAG deposition increases. Supplementary Figure 6 confirms that, as carbon allocation to TAG increased, the simulated net CO_2_ uptake rate decreased from 1.32 to 0.97 mol carbon/m^2^/day, while the energy inputs (photon uptakes) remain constant. This means that, from the same light energy inputs, 72.4% of the amount of CO_2_ that is fixed into sucrose can be fixed into TAG. Given a predicted daily TAG accumulation rate, the number of days until a desired amount of TAG per dry weight is reached can be calculated based on Equation 3 (see section “Materials and Methods”). Figure 5B shows the effect of change in carbon allocation on the predicted number of days until 1, 5, or 20% TAG (w/dw) are reached in mature leaves. For example, if 5% of net carbon assimilation was allocated to oil droplets, the time taken until a 20% TAG level is reached was predicted to be 18 days (Figure 5B). If 20% of net CO_2_ assimilation was allocated to oil droplets, then this level could be reached within 5 days. Altogether, it appears that if only a small shift in carbon allocation from assimilate toward oil droplet production can be achieved, substantial TAG accumulation should be possible within a month.

### Effect of Futile Cycling on Triacylglycerol Accumulation

Leaf lipase activities (e.g., TAG hydrolysis) and the capacity for oxidative degradation of fatty acids by peroxisomal β-oxidation can result in futile cycles of synthesis and degradation which diminish the rate of net TAG accumulation. In *iTJC1414x4*, cyclic synthesis and degradation of fatty acids and of TAG can be imposed onto the model by the numerical constraints shown in Equations 4, 5. We define cycling as the multiplication factor by which the rate of synthesis of fatty acids or TAG exceeds the rate at which fatty acids or TAG itself are deposited in TAG. Fivefold cycling of fatty acids (Equation 5), for example, means that the *de novo* synthesis of fatty acids is five times the amount of fatty acid that is processed and stored in TAG. All fatty acids that are produced but not stored can only enter the peroxisomal process of β-oxidation. Besides fatty acid cycling, TAG cycling means that newly synthesized TAG is hydrolyzed into glycerol and free fatty acids, which then can be re-utilized to synthesize TAG. This requires glycerol and free fatty acids to be activated to glycerol phosphate and CoA esters, both of which are ATP-driven processes (reactions “*GlycerolKinase*,” “*PalmitoylCoASynthesis*,” “*OleoylCoASynthesis*,” “*LinoleoylCoASynthesis*,” and “*LinolenoylCoASynthesis*”). Figure 4 compares a scenario of TAG synthesis from CO_2_ without cycling to a scenario where fivefold FA cycling takes place. At fivefold cycling, fatty acid biosynthesis is increased by 2.7-fold while the net CO_2_ uptake and TAG deposition rates decrease by 62 and 53%, respectively. It is also notable that the CBB cycle flux is predicted to markedly increase under the fivefold cycling condition (Figure 4). Degradation of fatty acids by β-oxidation transfers electrons onto oxygen and NAD^+^, and the produced NADH can contribute to the overall balance in reducing equivalents. Carbon from β-oxidation is predicted to be recovered as malate, and in the pyruvate/malate shuttle mechanism its production makes up for the reduced synthesis and transport of malate from MCs to BSCs (Figure 4).

Assuming all net CO_2_ was allocated toward oil droplets, we simulated the impact on the rate of TAG storage by levels of increasing futile cycling (Figures 5C,D). As seen in Figure 5C, as TAG cycling was increased up to about 50-fold, the rate of TAG deposition decreased only slightly. Notably, fatty acid cycling more strongly affects TAG deposition than TAG cycling. As can be deduced from the graphs in Figure 5C, a 50% reduction of the maximal rate of TAG deposition is obtained for about fivefold cycling of fatty acids, while about 100-fold TAG cycling is needed to reduce TAG deposition in the same way. This difference can be in part explained by the difference in re-synthesis cost. Re-synthesis of oleoyl-CoA from acetyl-CoA requires 9 ATP and 17 reducing equivalents, while re-cycling of one free oleic acid into TAG requires activation of the free fatty acid to the CoA ester by acetyl-CoA synthetase (EC 6.2.1.1) in coordination with adenylate kinase (EC 2.7.4.3), which comes at a net cost of 2 ATP. The effect of cycling on the daily net TAG deposition rate can be used to predict TAG accumulation over time (Equation 3). The time needed to reach a 20% TAG target increases as FA or TAG cycling increase (Figure 5D). These scenarios were considered again for a 100% allocation of CO_2_ net assimilation to oil droplets. As before, the time to reach target levels of TAG was much less substantially affected by TAG cycling than by FA cycling (Figure 5D).

### Combined Effects of Carbon Allocation and Futile Cycling

**FIGURE 6:**
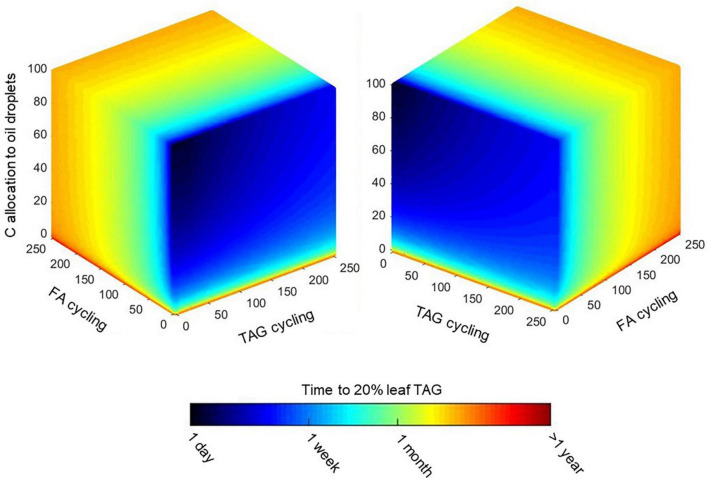
Concurrent effect of allocation and futile cycles on TAG accumulation. Expansion of the Figure 5 simulations to explore how simultaneous variations in net carbon allocation, FA cycling, and TAG cycling affect TAG accumulation in mature *Sorghum bicolor* leaves. Colors indicate the expected time needed to for stored TAG to occupy 20% (w/dw) of the leaf and are given on a log scale. Cycling units are mol TAG synthesis per mol TAG accumulated in oil droplets (TAG cycling) and mol fatty acid synthesis per mol fatty acid stored in oil droplets (FA cycling).

**FIGURE 7:**
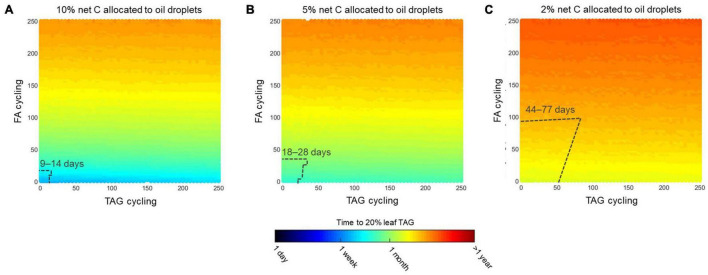
Effect of futile cycles at fixed carbon allocation ratios. Subsets of simulations presented in Figure 6 to illustrate how TAG and FA futile cycling together lead to changes in the time needed to accumulate 20% (w/dw) TAG in mature *Sorghum bicolor* leaves when **(A)** 10, **(B)** 5, or **(C)** 2% of net carbon is allocated to oil droplets. Colors indicate the expected time on a log scale. Dashed lines indicate a region close to the origin (0, 0) within which maximum flux limits in lipid metabolism are not exceeded. We set these flux limits based on a scenario where leaf biomass synthesis was maximized. In the delimited region simulations, the total fatty acid synthesis rate was less than 10-fold the synthesis rate in the growing leaf scenario. The range of days until 20% TAG is reached in the delimited region is given. Cycling units are mol TAG synthesis per mol TAG accumulated in oil droplets (TAG cycling) and mol fatty acid synthesis per mol fatty acid stored in oil droplets (FA cycling).

Both FA and TAG futile cycling can be imposed in combination with a carbon partitioning scenario. Figure 6 shows a factorial plot for simulating the three variables for up to 250-fold cycling for fatty acids and TAG. The time duration to reach 20% leaf TAG is shown in color coding. In agreement with the analysis in Figures 5, 6 shows how FA cycling has generally more substantial effects than TAG cycling across a range of carbon partitioning ratios. Although we show up to 250-fold futile cycling, model simulations with high cycling rates might be unlikely to occur in reality due to physiological limitations in flux capacity. To estimate limitations on fatty acid and TAG cycling fluxes, we predicted fatty acid biosynthesis rates when total leaf biomass is produced (Supplementary Table 3) under conditions of light flux, photorespiration, CO_2_ leakage and maintenance cost as defined above for the mature leaf reference state (Supplementary Table 2). This simulation predicted that fatty acids, while contributing to biomass formation, are newly formed at a rate of 0.11 mol/m^2^/day, which is equivalent to 12% of the net CO_2_ fixation rate. We now supposed that 10 times that rate (1.1 mol/m^2^/day) might define the upper limit in flux capacity for growing tissue or for mature tissue engineered to accumulate TAG. Figure 7 shows imposition of this flux capacity limit for discrete slices of Figure 6 at 10, 5, and 2% net carbon allocation to oil droplets. Simulation results where TAG and fatty acid biosynthesis operate below the upper limits are demarcated. For example, for 5% carbon allocation to oil droplets, maximal about 20-fold TAG and 25-fold FA cycling are feasible and within the demarcated area, the time to reach 20% TAG per leaf dry weight varies between 18 and 28 days (Figure 7B).

### Triacylglycerol Accumulation When Leaf Is Shaded Within the Canopy

The simulations on carbon allocation to TAG (Figures 5–7) were based on rates that can be expected when *S. bicolor* leaves receive full sunlight. Realistically, there are weather dependent reductions in sunlight, effects of temperature and draft conditions, effects of shading within the canopy, as well as reductions in metabolic capacity due to onset of senescence. Of these conditions we decided here to briefly explore the effect of shading, without detailed modeling of canopy effects. At 1 m depths into the canopy, *Z. mays* leaves have been measured to receive roughly 70% less light than fully exposed leaves (Dohleman and Long, 2009). Consequently, in our shade simulations, we constrained the MC and BSC to receive 70% less light, thus to reduce the total influx for light from 22.12 to 6.63 mol photons/m^2^/day. With these shade conditions, we simulated how the rate of TAG storage was affected if 0–100% of net CO_2_ assimilation was allocated to oil droplets, and there was up to 1,000-fold futile cycle activity. At 100% carbon allocation to oil droplets and without futile cycling, TAG could accumulate at 1.35 g/m^2^/day and reach the 20% yield target in less than 2 weeks (Supplementary Figure 7). If only 30% of net carbon assimilation is allocated to oil droplets or if there is 200-fold TAG cycling, then 20% TAG (w/dw) would be reached within a month.

## Discussion

As an emerging concept for the development of high energy renewable plant resources, engineering bioenergy crops to accumulate TAG in their vegetative tissues holds the promise of achieving high overall yields. Panicoid C_4_ grasses are generally known for their high productivity and potential for use as bioenergy crops. Of these, *S. bicolor* has excellent genomic resources (Carpita and McCann, 2008; Paterson, 2008; McCormick et al., 2018) and a diploid genome that has not experienced genome duplication since its diversion from the common ancestor with maize (Kim et al., 2014), which positions *S. bicolor* as a model for other panicoid C_4_ grasses with more complex polyploid genomes. *S. bicolor* is also amenable to genetic engineering. For example, *S. bicolor* leaves have been genetically engineered to over-express WRI, DGAT1, and oleosin and accumulated between 3 and 8.4% oil per leaf dry weight (Vanhercke et al., 2019a). To explore the theoretical capacity of *S. bicolor* to accumulate TAG, we derived a genome-referenced reconstruction of *S. bicolor* metabolism (*iTJC1414*). We put special emphasis on curation of primary metabolism, including metabolism relevant to fatty acid and TAG metabolism. We further present a C_4_-leaf diel FBA metabolic model (*iTJC1414x4*) that simulates cycles of day and night leaf metabolism to explore the theoretical capacity of *S. bicolor* to accumulate TAG in leaves.

Lipids are energy-dense plant products, which means that replacing sucrose by TAG as the photo-assimilatory end-product comes at a higher cost in energy cofactors that must be derived from the light-dependent reactions in chloroplasts. In literature related to plant lipid biofuels it is often pointed out that, on a weight basis, oil has more than twice the energy content of carbohydrate (Durrett et al., 2008; Ohlrogge and Chapman, 2011; Allen et al., 2015; Huang et al., 2016; Xu and Shanklin, 2016; Wan et al., 2017). Based on the fatty acid composition used in our model, sorghum leaf TAG is 2.39 as energy dense as sucrose (Table 2). Thus, one could infer that the biosynthetic cost raises by a similar factor. However, on a per carbon basis, TAG has about 1.4-fold the energy content of carbohydrate (Table 2). Similarly, the requirements in reducing equivalents based on the chemical balance are about 1.4 times higher in TAG (Table 2). In basic agreement with the considerations on energy content and chemical minimal demands, we find that in a C_3_ photosynthesis context the metabolic demands in ATP and NADPH are 1.3-fold and 1.49-fold higher for TAG, as compared to sucrose synthesis (Figure 2A). If photorespiration activity is considered, very similar ratios are found (Supplementary File 3). Evaluations of C_4_ photosynthesis in *iTJC1414x4* (Table 4, scenario 3) showed 1.37-fold higher demands in NADPH and slightly less than that of a difference in ATP cost. Overall, if done based on comparing energy density on per weight basis, the cost for photo-assimilation of CO_2_ into TAG will be overestimated.

The basic scenario for TAG biosynthesis in a leaf tissue shows that the generation of acetyl-CoA as a precursor of fatty acid biosynthesis is well-integrated with the CBB cycle (Figures 2A, 4). Indeed, experimental evidence has shown that the plant fatty acid synthesis pathway is co-localized with the CBB cycle to the chloroplast (Ohlrogge and Browse, 1995). Also, fatty acid biosynthesis in leaves has been shown to be strongly light dependent (Roughan and Ohlrogge, 1996; Bao et al., 2000). CO_2_ labeling kinetics suggest that CO_2_ is incorporated very fast into fatty acids while free acetic acid or sugars are unlikely direct pathway intermediates (Bao et al., 2000). Experimental evidence further suggests that acetyl-CoA in leaf chloroplasts is derived directly from CBB cycle intermediates *via* a chloroplast-localized PDHp (Murphy and Leech, 1977; Bao et al., 2000; Joyard et al., 2010). One advantage of proximity of the CBB cycle and fatty acid biosynthesis in one compartment should be very efficient re-fixation by RubisCO of CO_2_ emitted by PDHp (Figure 2A). Notably, the PDHp produces one mol NADH per mol acetyl-CoA (Figure 2A). While it is most commonly the case that the energy cofactors NADH and NADPH are dedicated toward use in cellular respiration and in reductive biosynthetic reactions, respectively, the NADH produced by PDHp can be assumed to be used entirely in fatty acid biosynthesis (Figure 2A). This is possible since one of the two reducing enzyme activities in the chloroplast fatty acid biosynthesis, enoyl-ACP reductase (EC 1.3.1.9), is considered to be specific to NADH, not NADPH (Slabas et al., 1986; Rafferty et al., 1994; Gonzalez-Thuillier et al., 2015). Besides the CBB cycle, the photosynthetic light reactions are co-localized with fatty acid biosynthesis as well. This suggests a tight coupling between photosynthetic ATP and NADPH supply and biosynthetic demands. It is widely considered that ATP and NADPH provided by the photosynthetic light reactions must match the metabolic demands to prevent photodamage and allow for optimal growth (Noctor and Foyer, 1998; Kramer and Evans, 2011; Walker et al., 2020). To explore the energy supply/demand balance, we defined a reference state for the mature sorghum leaf where photosynthesis ATP/NADPH supply matches the demands for synthesis of phloem-exported photo-assimilates. Relying on the electron transport model for C_4_ photosynthesis by Yin and Struik (2018), we consider the light energy supply to be defined in a realistic way. Specifically for sorghum, their study essentially predicts that BSCs almost entirely rely on CET and mostly lack protein components of LET (Yin and Struik, 2018). This means that most of the NADPH used in the CBB cycle in BSCs needs to be imported from MCs, as being the case in our simulation (Figure 3). Having therefore defined a reliable reference photosynthetic state, we could further predict that altering the CO_2_ assimilation product from phloem-exported photo-assimilates into TAG would lead to a sizeable surplus in photosynthetic ATP production (Table 4). An ATP surplus could stall the thylakoid ATP synthase, therefore likely leading to a build-up of the proton motive force that would restrict electron flow toward NADP^+^.

*iTJC1414x4* is a C_4_ metabolism diel model that allows simulation of the metabolism and resource allocation in a leaf over the diel cycle. We performed *in silico* simulations to evaluate the assumption that based on its capacity to photo-assimilate CO_2_, a mature sorghum leaf could potentially accumulate substantial levels of TAG in a short time. Critical for this would be that a sizeable fraction of the assimilated carbon can be diverted away from exported photo-assimilates to oil droplets and that the limiting effects of lipid turnover (futile cycles) can be mitigated. A range of possible scenarios of partitioning of carbon assimilation between oil droplets and photo-assimilates were simulated to predict net possible daily rates in TAG accumulation. We then expressed daily TAG accumulation rates as the number of days it would take to reach a 20% yield target. The 20% level would make production of *S. bicolor* biodiesel economically feasible (Huang et al., 2016). We determined that if only 5% of the net carbon assimilated would be partitioned toward oil droplet deposition and if there was moderate futile cycling, a 20% w/dw target could be reached in less than a month (Figure 7B). There are several insights that can be derived from this finding. If it is sufficient to divert only a small fraction of assimilated carbon into TAG synthesis, then the above-mentioned problem of energy imbalance for TAG synthesis would be less severe since, apart from TAG synthesis, most of the photosynthetic supply of ATP and NADPH would still be invested into sucrose synthesis and therefore mostly balance with the overall biosynthetic demands. Also, the finding that minimal carbon partitioning might be sufficient to accumulate TAG at substantial levels is useful for a retrospective assessment of reported efforts at engineering leaves to accumulate TAG. Keeping in mind that such past studies were done with different plant species and that TAG accumulation rates are typically not precisely measured, our modeling exploration shown in Figures 5–7 nevertheless suggests the following: if final TAG levels are found to be far below 20% and we assume the accumulation took place during a rather extended time period (>1 month), only minimal reallocation of primary photo-assimilate into TAG could have taken place. As a consequence of these considerations, it seems that engineering efforts should benefit from more detailed experimental characterization of transgenic events, like determination of carbon reallocation ratios by use of isotope tracers. Since our model simulations suggest that accumulation of TAG at substantial levels in leaf tissue is possible within a time frame of only several weeks, high-yield goals might be in reach if TAG biosynthesis is to take place only at the end of the growth cycle. This way, by engineering TAG synthesis to only be activated in the final stage of the crop life cycle, transgenic growth penalties could be largely avoided. The late onset of TAG synthesis could be achieved, for example, by chemical induction (Caddick et al., 1998) or using senescence related promoters (Kim et al., 2015; Vanhercke et al., 2017; Xu et al., 2020). For example, it is known for some species, that ectopic over-expression of the transcription factor WRI1 can cause perturbations in vegetative development (Marchieve et al., 2014). In case of potato plants it was found that, while expression of WRI1 in leaves has resulted in such effects, expression under a sensecence inducible promoter had less adverse affects, with TAG levels peaking at the sensecence stage of leaves (Xu et al., 2020). However, considering TAG accumulation in senescing leaves one also might have to address a number of senescense related effects, including lipid turn-over and degradation (Troncoso-Ponce et al., 2013).

Our model simulations explore a trade-off between photo-assimilates exported to the phloem and the synthesis and deposition of TAG in the leaf tissue. We consider export of sucrose and amino acids as the dominant route of assimilate flow in mature leaves. Starch is modeled as transitory pool (Figure 1) and is not included in the trade-off simulations. Similarly, the levels of leaf sugars are treated as invariably constant in our model. Of relevance here is that in various studies on engineering high oil content in leaves, differences in leaf starch or sugar levels between wild-type and transgenics have been found and that therefore leaf carbohydrates are regularly considered to be major sinks, competing with oil synthesis for photo-assimilates (reviewed in Xu and Shanklin, 2016; Vanhercke et al., 2019b). While a difference in carbohydrate levels might indicate that carbon allocation has been changed, we do not expect differences in leaf carbohydrate levels to be a quantitative indicator of changes in carbon allocation. This should become clearer by the following considerations. In our simulated mature leaf reference state, the sucrose export rate is 0.09 mol/m^2^/day while transitory starch is synthesized at a rate of 0.03 mol/m^2^/day (Supplementary Table 7, scenario 4). In weight units this sucrose export amounts to 30.8 g/m^2^/day. Considering the sorghum leaves to have a dry weight of 60 g/m^2^ in this study, the sucrose that is synthesized and exported per day can amount to 50% of the leaf’s dry weight. At the same time, sucrose levels that can be measured in a leaf are typically well below 10% w/dw and might undergo diurnal variation (Kalt-Torres et al., 1987; Ning et al., 2018; Liang et al., 2019; Mitchell et al., 2020). This means that, while leaf sucrose levels might be informative of the leaf’s sugar status, sucrose should be understood and modeled as a high turn-over pool. A change in sugar level is unlikely to reflect a change in photo-assimilation of in assimilate partitioning in a quantitative way. Our assertions on the dominance of assimilate export as a carbon sink can be supported by other studies. In maize leaves, CO_2_ assimilation during a normal day/night cycle has been found to result in generation of 45.5 g sucrose synthesis per m^2^ leaf area per day, from which 80% was exported (Kalt-Torres et al., 1987). Applying the leaf density that we used here (60 g dw/m^2^) to the study by Kalt-Torres et al. (1987), the daily sucrose export in maize leaves can amount to 60% of the leaf dw while the sucrose contents of the leaf were maximally at about 4% (w/dw) during the day. A later study confirmed that maize leaves at different developmental stages can export 80% or more of the photo-assimilated sugars (Liang et al., 2019). Overall, we conclude that in studies aimed at engineering plants to accumulate oils in vegetative tissue, the assessment of carbon allocation in leaves of the transgenics should benefit from measurements of overall the CO_2_ assimilation rate and from tracing this assimilate flow into major sinks.

In the context of this study, the simulated allocation scenarios are predominantly concerned with the assimilation and partitioning of carbon. For simulations relating to the mature leaf reference state, nitrogen assimilation takes place for the synthesis of amino acids that contribute to the exported photo-assimilates and to the oleosin associated with TAG. However, nitrogen assimilation occurs here at a relatively low rate. In a larger context, nitrate assimilation has been considered to be a major sink for photosynthetic energy in higher plants (Noctor and Foyer, 1998). For foliar nitrate assimilation, photosynthetic ATP production is expected to be in excess relative to the demands at the glutamine synthetase step (Noctor and Foyer, 1998), i.e., an ATP surplus is to be expected to occur. This can be demonstrated with *iTJC1414x4*. If the energy balance computations made for the mature leaf in Table 4 (scenario 3) are modified so that glutamate is the only photo-assimilate exported, nitrate and CO_2_ are assimilated at a ratio of 0.2:1 and the ATP surplus amounts to 1.69 ATP per CO_2_ fixed.

In this study we used a C_4_-leaf diel FBA metabolic model to explore the accumulation of TAG in the leaf. However, maximal energy density in the total above-ground harvested biomass of a bioenergy crop would be best accomplished if all above ground vegetative tissues would accumulate TAG. Therefore, extensions of our approach to a whole plant model need to be considered. Diel FBA models have been used in other plant studies to analyze resource transport and use in a whole-plant system (Gomes de Oliveira Dal’Molin et al., 2015), compare leaf metabolism in C_3_ and CAM plants during the day and at night (Cheung et al., 2014), and explore energetic limitations of product synthesis in C_4_ leaves (de Oliveira Dal’Molin et al., 2018). It is important to note that, although these models may connect and balance metabolism across different tissues and between day and night, multi-tissue and diel FBA modeling approaches do not necessarily make any prediction on the growth dynamics of a plant during its life cycle. Therefore, our current model cannot directly account for yield penalty effects that have been observed in plants engineered to constitutively accumulate TAG in green vegetative tissues (Alameldin et al., 2017; Vanhercke et al., 2019a). To model plant growth dynamics and address questions concerning potential yield penalties, whole-plant carbon partitioning and storage of photo-assimilate as TAG in other organs (e.g., stem), the diel steady-state FBA model presented here could be extended into a dynamic FBA (dFBA) model (Varma and Palsson, 1994). In a dFBA time series simulation of a multi-tissue model, each steady state modifies conditions like carbon allocation ratios of the next steady state. For example, dFBA has been used with a multiorgan barley model to investigate how decreases in photosynthesis due to leaf senescence limit the yield of seeds and other sink tissues (Grafahrend-Belau et al., 2013). In addition, Shaw and Cheung (2018) modeled *A. thaliana* with dFBA and estimated the total biomass and root-to-shoot ratios resulting from herbivory, shading, nitrogen availability, and other features that would have long-term impacts on carbon and/or nitrogen metabolism. By using dFBA to simulate TAG accumulation in vegetative tissues, the joint effects of plant development, leaf age, and other factors could be integrated.

## Conclusion

Our simulations of TAG accumulation in mature sorghum leaves suggest that economically feasible levels of TAG could be reached within weeks if only a small fraction of photosynthate is allocated to oil droplets and there is mild FA futile cycling. This means that engineering strategies aimed at activating TAG accumulation only at the end of the growth cycle might result in sufficiently high TAG levels to become economically feasible. Activating TAG accumulation late in development would also mean that the not well understood phenomenon of yield penalty could be avoided. Altogether, our study helps to establish a benchmark to measure metabolic engineering efforts aimed at TAG accumulation in vegetative tissues. Additional efforts to improve the metabolic engineering cycle should certainly also make use of quantitative analysis of metabolic phenotypes, which may include metabolomics and isotope tracer-based efforts at determining synthesis and turnover rates of TAG (Chu et al., 2020, 2022).

## Data Availability Statement

The original contributions presented in the study are included in the article/Supplementary Material, further inquiries can be directed to the corresponding author.

## Author Contributions

TC and JS conceived the project, reconstructed the genome-referenced, four cell, diel FBA model of sorghum, and co-wrote the manuscript. JS built the generic plant network for C_3_ photosynthesis and TAG synthesis, analyzed the photosynthetic sub-models, and aligned the Yin and Struik model to the FBA model. TC carried out the model simulations on carbon allocation and futile cycling. Both authors contributed to the article and approved the submitted version.

## Conflict of Interest

The authors declare that the research was conducted in the absence of any commercial or financial relationships that could be construed as a potential conflict of interest.

## Publisher’s Note

All claims expressed in this article are solely those of the authors and do not necessarily represent those of their affiliated organizations, or those of the publisher, the editors and the reviewers. Any product that may be evaluated in this article, or claim that may be made by its manufacturer, is not guaranteed or endorsed by the publisher.

## Footnotes

1
https://phytozome-next.jgi.doe.gov/

2
www.genome.jp/kegg/

3
www.plantcyc.org

4
www.python.org

5
https://www.gnu.org/software/glpk/

## Supplementary Material

Supplementary File 1 *Sorghum bicolor iTJC1414* single-cell metabolic model in SBML and Excel formats, and MATLAB scripts for running model simulations.

Supplementary File 2 *Sorghum bicolor iTJC1414x4* in SBML and Excel formats, and MATLAB scripts for running model simulations.

Supplementary File 3 Supplementary information on chemical balances, biochemical pathways, and energy budgets of photosynthesis.

Supplementary File 4 Input files for METATOOL analysis and model code for the cell type-specific electron transport model by Yin and Struik (2018).

## Abbreviations

*a*_*BS,M*_: BSC:MC ratio in light absorption
*f*_*LET,BS*_: fraction of BSC absorbed irradiance that is used for linear electron transport
*f*_*LET,M*_: fraction of MC absorbed irradiance that is used for linear electron transport
OP: oxidative phosphorylation
Φ_*CO2*_: absorbed quantum yield for CO_2_ uptake (mol CO_2_/mol photon).
