# Supplementary material for: Elucidation of Triacylglycerol Overproduction in the C4 Bioenergy Crop Sorghum bicolor by Constraint-Based Analysis
Source: Front Plant Sci. 2022 Feb 17;13:787265. doi: 10.3389/fpls.2022.787265 (PMC8892208; doi:10.3389/fpls.2022.787265)
Supplement: Supplementary File 3 — Supplementary information on chemical balances, biochemical pathways, and energy budgets of photosynthesis. [file Data_Sheet_3.pdf]

Supplementary File 3

- Supplementary information on chemical balances, biochemical pathways and energy budgets of photosynthesis
- Clark, T.J. & J. Schwender. (2022). Elucidation of triacylglycerol overproduction in the C4 bioenergy crop *Sorghum bicolor* by constraint-based analysis. *Frontiers in Plant Science*.

Contents:

1. Chemical and redox balances for conversion of CO_2 and water into reduced carbon compounds

2. Minimal biochemical pathways for CO_2 assimilation into sucrose and triacylglycerols by generic plant pathways
   - 2.1 Characterizing photosynthetic CO_2 assimilation using Elementary Flux Modes analysis
   - 2.2 Comparing of energy demands of biochemical pathways with chemical balances
   - 2.3 Assessing the ATP:reductant balance in photosynthesis
   - 2.4 Enzyme subsets in the minimal stoichiometric model

3. Calculations to determine investments of photosynthetic reducing equivalents and ATP into biosynthesis of sucrose and TAG in *iTJC1414x4*
   - 3.1 Pathways for light-driven production of reducing equivalents and ATP in *iTJC1414x4*
   - 3.2 Constraining linear electron transport (LET) relative to cyclic electron transport (CET) in *iTJC1414x4*
   - 3.3 Deriving sorghum-specific values for energy budget parameters (aBS,M , fLET,BS, fLET,M) from the Yin and Struik (2018) model
   - 3.4 Calculations for light-driven production of reducing equivalents and ATP from model simulations in *iTJC1414x4*

1. Chemical and redox balances for conversion of CO_2 and water into reduced carbon compounds

Here we derive general chemical mass and redox balances for a photosynthesis process resulting in a reduced carbon compound. As a reference, a general introduction into calculations of oxidation states in chemical equations can be found in Jurowski (2015). Consider the conversion of *x* moles CO_2 and *y* moles H_2O by a photosynthesis process into 1 mol of a reduced product $C_aH_bO_c$ and *z* moles O_2:

$$x\,\overset{+IV}{C}\overset{-II}{O_2} + y\,\overset{+I}{H_2}\overset{-II}{O} = 1\,\underset{\text{(product)}}{\overset{S}{C_a}\overset{+I}{H_b}\overset{-II}{O_c}} + z\,\overset{0}{O_2} \qquad (1)$$

The oxidation states of the elements are given in red and *S* is the average oxidation state of carbon in the product. Equation 1 shows that electrons are transferred from oxygen to carbon, i.e. only carbon and oxygen change redox state during the biosynthetic processes we consider here. Dependent on its elemental composition (*a*, *b*, *c*) the product can be various carbohydrates and carbohydrate polymers, fatty acid compounds (e.g., triacylglycerol), terpenoids or other hydrocarbon compounds.

To resolve *x*, *y*, and *z* in dependence of *a*, *b*, and *c* we re-write equation (1) to show the balanced equations for the elements:

$$\begin{array}{cccc} CO_2 & H_2O & C_aH_bO_c & O_2 \end{array}$$
$$\begin{pmatrix} -x & & a & \\ & -2y & b & \\ -2x & -y & c & 2z \end{pmatrix} = \begin{pmatrix} 0 \\ 0 \\ 0 \end{pmatrix}, \qquad (2)$$

with rows 1, 2 and 3 being the elemental balances for C, H and O, respectively. The first two rows solve to $x = a$ and $y = ½b$. If then substituted into the third row, we obtain $z = (2a + ½b - c)/2$. This means that equation (1) can be re-written as a function of the elemental composition of the product (*a*, *b*, *c*):

$$a\overset{+IV\,-II}{CO_2} + ½b\overset{+I\,-II}{H_2O} = 1\underset{\text{(product)}}{\overset{S\,+I\,-II}{C_aH_bO_c}} + \frac{2a + b/2 - c}{2}\overset{0}{O_2} \qquad (3)$$

Now we express the redox state *S* as a function of *a*, *b* and *c*. Considering only uncharged products, the redox balance for $C_aH_bO_c$ is $Sa + (+1)b + (-2)c = 0$, which can be solved for *S*:

$$S = \frac{2c - b}{a}\ . \qquad (4)$$

Now we consider the number of electrons transferred in the process. Oxygen changes its redox state from -2 to 0. Since two electrons are removed from each oxygen atom, 4 electrons are removed from each O_2. The 4 electrons multiplied with the stoichiometric coefficient for O_2 in equation (3) ((2a+ ½b – c)/2), means that:

$$electrons\ removed\ from\ oxygen = 4a + b - 2c$$

To confirm this balance one can derive the electron transfer for carbon. Carbon changes its oxidation state from +4 (in CO_2) to *S*. Using coefficient *a* from equation 3, the molar amount of electrons transferred is:

$$electrons\ transferred\ to\ carbon = 4a - Sa$$

By substituting *S* with (2*c* – *b*)/*a* (Equation 4) we obtain:

$$electrons\ transferred\ to\ carbon = 4a + b - 2c, \qquad (5)$$

which is the same quantity of electrons that are removed from oxygen above. Altogether, 4*a* + *b* – 2*c* electrons are transferred from oxygen onto carbon in the process. This is thought to be mediated by reducing cofactors like NADH or NADPH. 1 mol NAD(P)H transfers two electrons, which is sometimes referred to as "reducing equivalent." Using Equation 5, the number of reducing equivalents involved in the overall process is half the number of transferred electrons:

$$reducing\ equivalents = 2a + \frac{b}{2} - c \qquad (6)$$

The procedure above to derive *x*, *y*, *z*, *S* and reducing equivalents can be applied to various possible product compounds ($C_aH_bO_c$) listed in Table SF2-1. For example, triolein ($C_{57}H_{104}O_6$) can be produced with the balance:

$$57\,CO_2 + 52\,H_2O = 80\,O_2 + 1\,triolein \quad (7)$$

In the process the electron transfer can be quantified as 160 reducing equivalents (equation 6), which is 2.81 reducing equivalents per fixed CO_2 (Table SF2-1).

Table SF2-1. Chemical balances for the formation of carbon-reduced products from CO_2 and water according to Equation 3. For each composition (formula) the stoichiometric coefficients *x*, *y* and *z* are listed under CO_2, H_2O and O_2, respectively.

Product	formula	CO_2	H_2O	O_2	required reducing equivalents	required reducing equivalents "H_2" per CO_2	average oxidation state of carbon (*S*)	Energy density (kJ/mol)[1]	Energy density (kJ/mol)[2]
glyoxylate	$C_2H_2O_3$	2	1	1	2	1.000	2.000	-459.80	n/a
citric acid	$C_6H_8O_7$	6	4	4.5	9	1.500	1.000	-2048.20	-1960.6
glucose	$C_6H_{12}O_6$	6	6	6	12	2.000	0.000	-2758.80	-2805.00
D-ribose	$C_5H_{10}O_5$	5	5	5	10	2.000	0.000	-2299.00	-2347.59
cellulose/starch	$C_6H_{10}O_5$*	6	5	6	12	2.000	0.000	-2717.00	n/a
sucrose	$C_{12}H_{22}O_{11}$	12	11	12	24	2.000	0.000	-5475.80	-5643.40
acetate	$C_2H_4O_2$	2	2	2	4	2.000	0.000	-919.60	-872.4
lignin	$C_{10.3}H_{13.9}O_{3.3}$	10.3	6.8	12.1	24.1	2.335	-0.670	-5324.90	n/a
trilinolenin	$C_{57}H_{92}O_6$	57	46	77	154	2.702	-1.404	-34108.80	-34108
sorghum leaf TAG[3]	$C_{56.2}H_{94.8}O_6$	56.2	47.4	76.9	153.8	2.737	-1.473	-34125.52	n/a
trilinolein	$C_{57}H_{98}O_6$	57	49	78.5	157	2.754	-1.509	-34861.20	-34555.70
triolein	$C_{57}H_{104}O_6$	57	52	80	160	2.807	-1.614	-35613.60	-35099.60
laurate	$C_{12}H_{24}O_2$	12	12	17	34	2.833	-1.667	-7607.6	-7423.7
tripalmitin	$C_{51}H_{98}O_6$	51	49	72.5	145	2.843	-1.686	-32353.2	-31605.9
tristearin	$C_{57}H_{110}O_6$	57	55	81.5	163	2.860	-1.719	-36366.00	-35806.70
stearate	$C_{18}H_{36}O_2$	18	18	26	52	2.889	-1.778	-11620.40	-11290.80
1-dodecene	$C_{12}H_{24}$	12	12	18	36	3.000	-2.000	-8025.60	-7925.90
ethanol	$C_2H_6O_1$	2	3	3	6	3.000	-2.000	-1379.40	-1368.00
octane	C_8H_{18}	8	9	12.5	25	3.125	-2.250	-5601.20	-5430.00
methane	CH_4	1	2	2	4	4.000	-4.000	-919.60	-890.00

* cellulose monomer
[1] estimate for heat of combustion derived from elemental composition according to Schmidt-Rohr (2015)
[2] as available, empirical values for enthalpy of combustion ($\Delta_cH°$) were taken from http://webbook.nist.gov/
[3] fatty acid composition used in *iTJC1414x4* (13.33% C16:0, 6.67% C18:1, 20% C18:2, 60% C18:3)

Table SF2-1 lists examples for organic acids, sugars, fossil fuel hydrocarbon compounds and triacylglycerols. Across the listed compounds there is excellent correlation between the number of required reducing equivalents and the energy density (heat of combustion) (Figure SF1). This becomes clear when the chemical transformation of CO_2 and water into a carbon-reduced product and oxygen (Equation 1) is considered in reverse as a combustion process. Enthalpy of combustion at standard condition (Δ_cH^o) can be estimated from the amount of oxygen reduced to H_2O. For a wide range of organic

compounds, $\Delta_c H^o$ is well approximated by a value of -444.56 KJ/mol oxygen consumed (Battley, 1999). This also applies to the compounds considered here (see R^2 value in Figure SF1). The slope obtained in Figure SF1 differs from the reported value by about 1 %. Therefore, in the synthesis direction, the amount of oxygen produced or required reducing equivalents (Table SF2-1) predicts very well the energy content of the product. This also implies that in a photosynthesis biochemical pathway the investments in reducing equivalents (NADPH) accounts for most of the energy content in the product. One might wonder if biosynthetic demands in ATP should not represent an investment into energy content of the product as well. The role of ATP in biosynthetic pathways can be understood as facilitating the formation of activated intermediates like acetyl-CoA and activated amino acids at physiologically relevant concentrations (Hanson, 1989). The role of ATP produced by the photosynthetic light reactions can then be understood as an energetic driving force for the reduction of CO_2 into carbon reduced compounds.

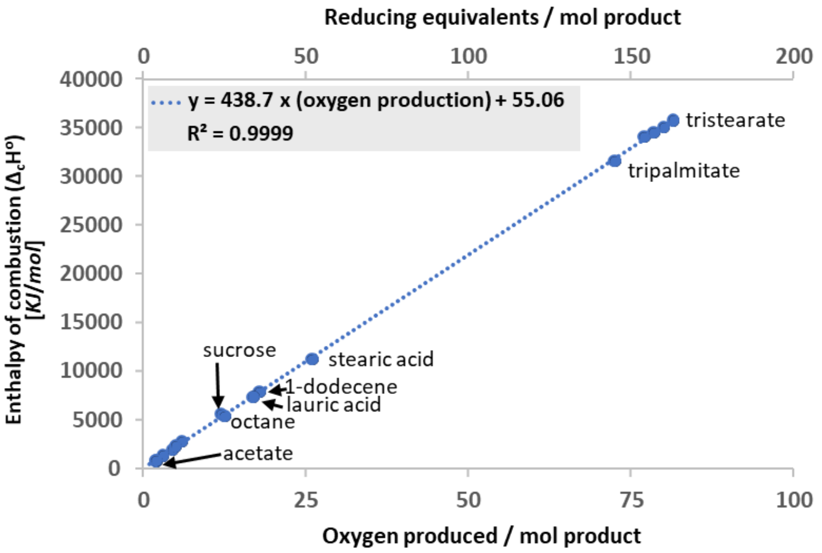

Figure SF1: Relationship between enthalpy of combustion (heat of combustion) of photo assimilatory product and oxygen produced. Values are taken from Table SF2-1 (only empirical $\Delta_c H^o$ values are plotted). The upper horizontal axis shows requirements in reducing equivalent which, according to equations 2 and 6 above, is ½ oxygen produced.

2. Minimal biochemical pathways for CO_2 assimilation into sucrose and triacylglycerols by generic plant pathways

2.1 Characterizing photosynthetic CO_2 assimilation using Elementary Flux Modes Analysis

In addition to the chemical balances, stoichiometries for the photosynthetic conversion of CO_2 into reduced carbon compounds were derived based on a set of minimal plant specific biochemical pathways. To calculate cofactor burdens for assimilation of CO_2 into sucrose or triacylglycerol (TAG), we derived a non-compartmentalized minimal stoichiometric network (N44) based on canonical plant pathways. CO_2 concentration mechanisms of C_4 photosynthesis are not represented. A schematic representation of the model is shown in Figure SF2. The model consists of 13 canonical reaction steps of the Calvin-Benson-Bassham (CBB) cycle (Sharkey and Weise, 2012), 11 reaction steps involved in photorespiration (Peterhansel et al., 2010), 4 reactions for glycolysis to convert 3-phosphoglycerate to acetyl-CoA and 6 reactions for sucrose synthesis from fructose 6-phosphate via sucrose phosphate synthase (Stitt et al., 1987; Kruger, 1997). Also, 6 reactions for TAG synthesis are present of which fatty

acid synthesis itself is the summed stoichiometry of 45 reactions of the chloroplast fatty acid biosynthetic process (see model file "Suc_TAG_stoichiometry.txt") which can be referred to in Li-Beisson *et al.* (2013). Acetyl-CoA entering *de novo* fatty acid biosynthesis is thought to be derived directly from CBB-cycle intermediates via pyruvate kinase and the pyruvate dehydrogenase complex (Joyard et al., 2010). RubisCO was defined as two distinct reactions, one concerning the carboxylation or ribulose 1,6 bisphosphate (reaction name "RubisC") and one as having carboxylation and oxygenation with substrates CO_2 and O_2, respectively, in the ratio of 3:1 ("RubisCO"). No oxidative pentose phosphate pathway or tricarboxylic acid cycle reactions were added. To avoid minor cofactor imbalances for TAG synthesis, a NADH/NADPH transhydrogenase functionality had to be added. As detailed below under "enzyme subsets", enzyme subsets analysis confirms that we have obtained a minimal model where any reaction is essential at least for one of the end products sucrose and TAG.

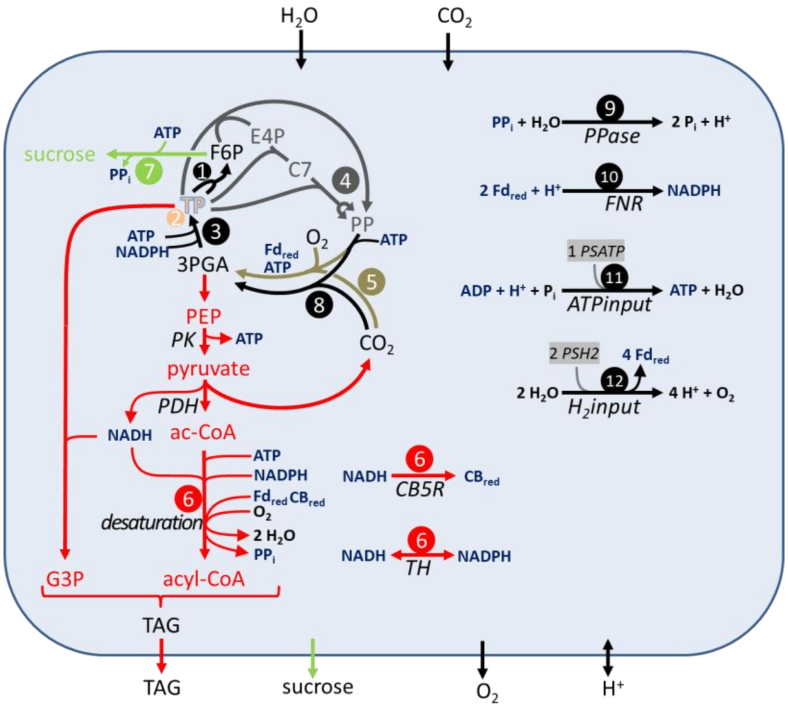

Figure SF2: Schematic overview of minimal pathway model with 44 reactions, used to derive stoichiometries for the conversion of CO_2 and water to sucrose or TAG by an oxygenic photosynthesis process. The model is plant typical with respect to the Calvin Benson Bassham cycle, the generation of acetyl-CoA by pyruvate dehydrogenase complex and the lumped stoichiometries of fatty acid biosynthesis with desaturation steps. Enzyme subsets (refer to Table SF2-4) are numbered and indicated by color coding. A transhydrogenase reaction (TH) was added to correct small imbalances in NADH production and consumption in case of TAG synthesis. See supplemental files "Suc_TAG_stoichiometry.txt" and "FAS_stoichiometry.txt". CO_2, water, O_2, sucrose and TAG (fatty acid composition of *iTJC1414x4*) can be exchanged with the environment. *PSATP*, *PSH2* are dummy metabolite species used to account total energy cofactor demands in the conversion of CO_2 and water to products. **Abbreviations:** AcCoA, acetyl-Coenzyme A; ATPsyn, ATP synthesis by chloroplast ATPase; C5, pentose phosphates; C7, sedoheptulose phosphates; CB5R, cytochrome B5 reductase; CB_{red}, reduced cytochrome B5; E4P, erythrose-phosphate; F6P, fructose-6-phosphate; Fd_{red}, reduced ferredoxin; FNR, ferredoxin-NADP reductase; G3P, glycerol-3-phosphate; PDH, pyruvate dehydrogenase complex; PEP, phosphoenolpyruvate; 3PGA, 3-phosphoglycerate; PK, pyruvate kinase; PP_i, pyrophosphate; PP, pentose phosphates; PPase, inorganic pyrophosphatase; *PSATP*, *PSH2*, dummy metabolite species used to account total energy cofactor demands in the total conversion balance; TAG, triacylglycerol; TH, transhydrogenase reaction; TP, triose phosphates.

Possible conversions of the biochemical network were limited by allowing only CO_2, O_2, H_2O, sucrose and TAG to be exchanged with the environment. In analogy to the chemical minimum calculations above, the flux modes computations allow obtaining the overall chemical balance for sucrose or TAG synthesis along with the amount of photosynthetic ATP and reducing equivalents that need to be invested in the process (Figure SF2). Given unidirectionality of key biosynthetic reactions, all computed elementary flux modes (Pfeiffer et al., 1999; Schuster et al., 1999, 2000) represent flux states with net conversion of CO_2 and H_2O into sucrose or TAG. The model file "Suc_TAG_stoichiometry.txt" depends on fatty acid biosynthesis stoichiometries being generated with file "FAS_stoichiometry.txt". The model files were executed with METATOOL version 4.3 (Pfeiffer et al., 1999; Schuster et al., 1999, 2000) on a Windows platform to obtain all possible balanced conversions. Altogether, the network combines plant typical enzyme reactions at a minimum required to perform the desired net conversions likely to occur in an autotrophic tissue of a C_3 plant.

Below are the main outputs from three METATOOL runs (ELEMENTARY MODES section). Each time the fatty acid reaction (obtained from model "FAS_stoichiometry.txt", Supplementary File 4) is replaced to represent a different fatty acid composition in TAG:

TAG = sorghum TAG (13.33% C16:0, 6.67% C18:1, 20% C18:2, 60% C18:3):

```
1A: 12 CO2 + 11 H2O + 37 PSATP + 24 PSH2 = 12 O2 + SUC
2A: 60 CO2 + 55 H2O + 305 PSATP + 192 PSH2 = 60 O2 + 5 SUC
3A: 562 CO2 + 474 H2O + 2248 PSATP + 1674 PSH2 = 769 O2 + 10 TAG
4A: 2810 CO2 + 2370 H2O + 19520 PSATP + 13338 PSH2 = 3845 O2 + 50 TAG
```

TAG = tristearate (C18:0):

```
1B: 114 CO2 + 110 H2O + 456 PSATP + 326 PSH2 = 163 O2 + 2 TAG
2B: 570 CO2 + 550 H2O + 3960 PSATP + 2638 PSH2 = 815 O2 + 10 TAG
3B: 12 CO2 + 11 H2O + 37 PSATP + 24 PSH2 = 12 O2 + SUC
4B: 60 CO2 + 55 H2O + 305 PSATP + 192 PSH2 = 60 O2 + 5 SUC
```

TAG = trilinolenin (C18:3):

```
1C: 12 CO2 + 11 H2O + 37 PSATP + 24 PSH2 = 12 O2 + SUC
2C: 60 CO2 + 55 H2O + 305 PSATP + 192 PSH2 = 60 O2 + 5 SUC
3C: 57 CO2 + 46 H2O + 228 PSATP + 172 PSH2 = 77 O2 + TAG
4C: 285 CO2 + 230 H2O + 1980 PSATP + 1364 PSH2 = 385 O2 + 5 TAG
```

In each output there are two modes for sucrose (SUC) production and two for TAG production,. The sucrose outputs are each time the same. The overall demands in ATP and reducing equivalents are obtained via the dummy metabolites "*PSATP*" and "*PSH2*". The outputs labeled in red are modes without photorespiration while in the other modes RubisCO carboxylation/oxygenation taking place at a ratio 3:1.

2.2 Comparing of energy demands of biochemical pathways with chemical balances

Table SF2-2 compares chemical balances from Table SF2-1 with the pathway balances above. Apart from the energy cofactor demands, all net biochemical pathway balances for sucrose and TAG synthesis fully agree with the chemical balances (Table SF2-2). For sucrose synthesis under the non-photorespiration condition, both the chemical and the biochemical pathway reducing equivalent requirements are the same. The same applies to tristearin as a product (Table SF2-2) while for production of trilinolenin the pathway version requires 172 moles reducing equivalents vs. 154 by chemical balance (Table SF2-2). The difference of 18 reducing equivalents will be understood when we consider that there are 9 double bonds in the fatty acid chains of trilinolenin. The formation of each double bond consumes two extra reducing equivalents. This is due to the plant specific enzyme reaction mechanism of acyl chain desaturation which transfers two electrons from the acyl chain (formerly invested in the fatty acid elongation process) plus one extra reducing equivalent back to one molecule of oxygen (Shanklin and Cahoon, 1998). We therefore find that, due to the specific mechanisms of fatty acid desaturation, the investment in reducing equivalents in trilinolenin biosynthesis is 10.5% (100% x 18/172) above the

chemical minimum. In case of TAG with composition as in *iTJC1414x4* this over-investment is 8.1% (Table SF2-2, Δ value). This additional expense can be avoided in bacterial fatty acid biosynthesis, essentially by skipping the enoyl-ACP reduction step during fatty acid elongation (Chan and Vogel, 2010). Photorespiration is a similar case of a pathway structure where reducing equivalents that were initially obtained by photolysis of water are transferred back to oxygen (Bauwe et al., 2010). Here this takes place via the oxygenation of ribulose 1,5 bisphosphate (RubisCO) and the combined activities of glycolate oxidase (EC 1.1.3.15) and catalase (EC 1.11.1.6). The photorespiration-associated over-investment in reducing equivalents is 60 %, 61.8 %, 77.1 % and 73.4 % for sucrose, tristearin, trilinolenin and sorghum TAG, respectively (Table SF2-2).

Table SF2-2: Chemical balances for the formation of carbon-reduced products by C_3 photosynthesis. Per mol product, net consumption of CO_2 and H_2O and production of O_2 are compared along with energy cofactor requirements. Reducing equivalent demands are identical between chemical balances and pathway balances, unless indicated: "+" indicates over-investment in reducing equivalents relative to the chemical minimum (Table SF2-1).

Product	formula	CO_2	H_2O	O_2	required reducing equivalents[1]	Required ATP[1]	
sucrose	$C_{12}H_{22}O_{11}$	-12	-11	12	24	-	(Chemical balance)
		-12	-11	12	24	37	(no photorespiration)
		-12	-11	12	38.4 (+60%)	61	(photorespiration)
tristearin	$C_{57}H_{110}O_6$	-57	-55	81.5	163	-	(Chemical balance)
		-57	-55	81.5	163	228	(no photorespiration)
		-57	-55	81.5	263.8 (+61.8%)	396	(photorespiration)
trilinolenin	$C_{57}H_{92}O_6$	-57	-46	77	154	-	(Chemical balance)
		-57	-46	77	172 (+10.5%)	228	(no photorespiration)
		-57	-46	77	272.8 (+77.1%)	396	(photorespiration)
sorghum leaf TAG[3]	$C_{56.2}H_{94.8}O_6$	-56.2	-47.4	76.9	153.8	-	(Chemical balance)
		-56.2	-47.4	76.9	167.4 (+8.1%)	224.8	(no photorespiration)
		-56.2	-47.4	76.9	266.76 (+73.4%)	390.4	(photorespiration)

1, per mol of product; 3, composition used in *iTJC1414x4* (13.33% C16:0, 6.67% C18:1, 20% C18:2, 60% C18:3)

2.3 Assessing the ATP:reductant balance in photosynthesis

Table SF2-3 expresses results from Table SF2-2 on basis of moles CO_2 assimilated. Also, the ratio of ATP demand to demand in reducing equivalent is shown (Table SF2-3). These ratios are relevant since photosynthetic production of ATP and NADPH is known to be tightly coupled and there might be limited flexibility of the photosynthetic apparatus to adapt its output to changing demands (Noctor and Foyer, 1998). In *iTJC1414x4*, on the supply side of energy cofactors, the production of ATP and NADPH is coupled based on the combined stoichiometries of the photosynthetic light reactions. In each cell model, if only linear electron transport occurs, the produced ratio of ATP/NADPH is 1.5 (see below section 3.1). If light drives both linear and cyclic electron transport, the ratio can be above 1.5. Curiously, Table SF2-3 shows that for TAG biosynthesis and without photorespiration the ratio of demands in ATP/NADPH is below 1.5. If in *iTJC1414x4* the production of ATP and NADPH is forced to occur at a ratio of 1.5 or above, then, in order to meet NADPH demands, ATP must be overproduced. The surplus in ATP cannot be used for the biosynthetic process. To avoid an imbalance the surplus ATP needs to dissipate, which in FBA simulations materializes in futile cycles with a net hydrolysis in ATP or the use of sub-optimal pathways. In *iTJC1414x4* we can quantify this ATP surplus by adding a dummy reaction "ATP surplus" (ATP + H_2O → ADP + phosphate). Maximization of the ATP surplus reaction to the model simulation procedure reveals how much ATP is being "wasted" in the network (see main text, methods). In addition, in simulations with

iTJC1414x4 we will apply constraints on the distribution of photon flux between the BS- and M-cells and between linear and cyclic electron flow. In this case, the ATP:NADPH production ratios are fixed to specific ratios in BS- and M-cells, respectively.

Table SF2-3: Biosynthetic demands in ATP and reducing equivalents. Based on values in Table SF2-2.

product	***PSATP* (per CO_2)**	***PSH_2* (per CO_2)**	***ATP/PSH_2***
No photorespiration (RubisCO carboxylation and oxygenation operating at a 1:0 ratio)			
sucrose	3.083	2.000	1.542
sorghum leaf TAG [1]	4.000	2.979	1.343
tristearate	4.000	2.860	1.399
trilinolenate	4.000	3.018	1.326
ratio triacylglycerols / sucrose	1.297	1.430-1.509	
Photorespiration (RubisCO carboxylation and oxygenation operating at a 3:1 ratio)			
sucrose	5.083	3.200	1.588
sorghum leaf TAG [1]	6.947	4.747	1.463
tristearate	6.947	4.628	1.501
trilinolenate	6.947	4.786	1.452
ratio triacylglycerols / sucrose	1.367	1.446-1.496	

1, composition used in *iTJC1414x4* (13.33% C16:0, 6.67% C18:1, 20% C18:2, 60% C18:3)

2.4 Enzyme subsets in the minimal stoichiometric model

With the computation of Elementary Flux Modes, the network was also broken down by enzyme subsets, which are sets of network reactions that, for all possible flux modes, always operate together at fixed flux proportions (Pfeiffer *et al.*, 1999). Table SF2-4 lists the 12 enzyme subsets obtained from the METATOOL output. There are four enzyme subsets with more than two reactions. They represent the regeneration of ribulose 1,6-bisphosphate from triose-phosphates and fructose-6-phosphate (mode 4), carboxylation/oxygenation (3:1) of ribulose-1,6-bisphosphate and regeneration of 3-phosphoglycerate (mode 5), TAG synthesis from 1,3-dihydroxyacetone-phosphate and 3-phosphoglycerate (mode 6) and sucrose synthesis from fructose-6-phosphate (Table SF2-4).

Table SF2-4: Enzyme subsets with their coupled stoichiometries for the minimal network producing sucrose and TAG from CO_2 assimilation (see Figure SF2). All subsets are active for formation of sucrose or TAG, except for modes 6 and 7. TAG, sucrose, H_2O and O_2 can be exchanged with the environment (external species). *PSH_2* and *PSATP* are external dummy metabolite species that allow to account for demands in photosynthetic inputs. Reversibility / irreversibility of subsets is indicated by "⇔" and "➔".

**subset**	**Enzyme subset (number of reactions in subset)**	**Subset stoichiometry**
1	Fructose-bisphosphate aldolase and -bisphosphatase (2)	**1** 1,3-dihydroxyacetone-phosphate + **1** glyceraldehyde-3-phosphate + **1** H_2O ➔ **1** fructose-6-phosphate + **1** Pi
2	Triose phosphate isomerase (1)	**1** 1,3-dihydroxyacetone-phosphate ⇔ **1** glyceraldehyde-3-phosphate
3	glyceraldehyde 3-phosphate dehydrogenase, phosphoglycerate kinase (2)	**1** ADP + glyceraldehyde-3-phosphate + **1** NADP + **1** P_i ⇔ **1** ATP + **1** NADPH + **1** 3-phosphoglycerate + **1** H^+

4	regeneration of RuBP (7)	**1** ATP + **⅓** 1,3-dihydroxyacetone-phosphate + **⅓** fructose-6-phosphate + **⅔** glyceraldehyde-3-phosphate + **⅓** H_2O ➔ **1** ADP + **⅓** P_i + **1** ribulose 1,6-bisphosphate + **3** H^+
5	Photorespiration (11)	**2** ATP + **8** ribulose-1,6-bisphosphate + **5** CO_2 + **3** O_2 + **7** H_2O + **2** ferredoxin(reduced) ➔ **2** ADP + **3** P_i + **15** 3-phosphoglycerate + **2** ferredoxin(oxidized) + **15** H^+
6	TAG synthesis (10)	**3** ATP + **1** 1,3-dihydroxyacetone-phosphate + **25.8** NADPH + **5.2** ferredoxin(reduced) + **26.6** 3-phosphoglycerate + **6.8** O_2 + **57.6** H^+ ➔ **3** ADP + **25.8** NADP + **5.2** ferredoxin(oxidized) + **24.6** P_i + **3** pyrophosphate + **26.6** CO_2 + **1** *TAG* + **36.2** H_2O
7	sucrose synthesis (6)	**1** ATP + **2** fructose-6-phosphate + **1** H_2O ➔ **1** ADP + **1** P_i + **1** pyrophosphate + **1** *sucrose*
8	RubisCO carboxylase (1)	**1** ribulose-1,6-bisphosphate + **1** CO_2 + **1** H_2O ➔ **2** 3-phosphoglycerate + **2** H^+
9	Inorganic pyrophosphatase (1)	**1** pyrophosphate + **1** H_2O ➔ **2** P_i + **1** H^+
10	ferredoxin-NADP+ reductase, EC 1.18.1.2 (1)	**2** ferredoxin(reduced) + NADP + **1** H^+ ➔ **2** ferredoxin(oxidized) + NADPH
11	ATPinput	**1** ADP + **1** P_i + **1** *PSATP* + **1** H^+ ➔ **1** ATP + **1** H_2O
12	H_2Input	**4** ferredoxin(oxidized) + **2** H_2O + **2** *PSH_2* ➔ **4** ferredoxin(reduced) + **1** O_2 + **4** H^+

Subsets: 1: Fructose bisphosphatase, EC 3.1.3.11; fructose bisphosphate aldolase, EC 4.1.2.13. **2:** Triosephosphate isomerase, EC 5.3.1.1. **3:** glyceraldehyde 3-phosphate dehydrogenase, EC 1.2.1.13; phosphoglycerate kinase, EC 2.7.2.3. **4:** ribose-5P isomerase, EC 5.3.1.6; ribulose-5P epimerase, EC 5.1.3.1; transketolase (I,II), EC 2.2.1.1; Aldolase (SBP), EC 4.1.2.13; Sedoheptulose bisphosphatase, EC 3.1.3.37; Phosphoribulokinase, EC 2.7.1.19. **5:** RubisCO carboxylase/oxygenase with carboxylase:oxygenase = 3:1, EC 4.1.1.39; 2-phosphoglycolate phosphatase, EC 3.1.3.18; glycolate oxidase EC 1.1.3.15; Katalase, EC 1.11.1.6; Ser:Glyox aminotransferase, EC 2.6.1.45; Glu:Glyoxylate aminotransferase, EC 2.6.1.4; Glycine decarboxylase activities + serine hydroxymethyltransferase EC 1.4.4.2/2.1.2.10/1.8.1.4/2.1.2.1; NAD hydroxypyruvate reductase EC 1.1.1.29; Glycerate kinase EC 2.7.1.31; glutamate synthase EC 1.4.7.1; Glutamine synthetase, EC 6.3.1.2. **6:** Phosphoglyceromutase, EC 5.4.2.1; enolase, EC 4.2.1.11; pyruvate kinase, EC 2.7.1.40; pyruvate dehydrogenase complex EC 1.2.4.1/2.3.1.12/1.8.1.4; glycerol-3-phosphate dehydrogenase (NAD+), EC 1.1.1.8; lumped reactions for fatty acid biosynthesis and TAG synthesis; Adenylate kinase, EC 2.7.4.3; generic NADH/NADPH transhydrogenase; cytochrome-b5 reductase, EC 1.6.2.2. **7:** glucose-6-phosphate isomerase, EC 5.3.1.9; phosphoglucomutase, EC 5.4.2.2; UDP glucose pyrophosphorylase, EC 2.7.7.9; sucrose-phosphate synthase, EC 2.4.1.14; sucrose-phosphate phosphatase, EC 3.1.3.24, Adenylate kinase, EC 2.7.4.3. **8:** RubisCO carboxylase/oxygenase (carboxylase:oxygenase 1:0), EC 4.1.1.39. **9:** inorganic diphosphatase; EC 3.6.1.1. **10**: ferredoxin-NADP+ reductase, EC 1.18.1.2. **11, 12**: Pseudo-reactions for generation of ATP and reducing equivalents.

3. Calculations to determine investments of photosynthetic reducing equivalents and ATP into biosynthesis of sucrose and TAG in iTJC1414x4

3.1 Pathways for light-driven production of reducing equivalents and ATP in *iTJC1414x4*

The photosynthetic ATP and NADPH yields in *iTJC1414x4* were defined in reference to Kramer and Evans (2011). According to Table 1 in Kramer and Evans (2011), linear electron transport (LET) produces ATP and NADPH at a fixed ratio with the following stoichiometries: 4 absorbed photons result in 1 reducing equivalent (NADPH) and 6 H^+ are transported. If H^+-ATPase is operating at 4 H^+/ATP, then 1.5 ATP are produced. For cyclic electron transport (CET), Kramer and Evans (2011) discuss four different possible pathway variants. We adopted the stoichiometry common to pathways 2, 3 and 4 in Table 1 of Kramer and Evans (2011), which are slightly less efficient than a pathway including the NADPH dehydrogenase complex. In this stoichiometry, for every 2 photons absorbed in PSI, 4 H^+ are transported. With H^+-ATPase operating at 4 H^+/ATP, 1 ATP is produced. Our assumptions here on LET and CET also conform with assumptions made by Yin and Struik (2018). In addition to the direct production of ATP and NADPH by the photosystems, it is known that under certain conditions the transfer of electrons from chloroplast LET to mitochondria takes place to allow generation of extra ATP in mitochondria (Pastore et al., 2003; Kramer and Evans, 2011). In *iTJC1414x4*, the transfer of electrons from chloroplast LET to the respiratory chain in the mitochondrion is possible by reactions that comprise the "malate valve" shuttle system (Selinski and Scheibe, 2019). The stoichiometric coupling between photon absorption, electron flow, photophosphorylation as well as between electron flow and mitochondrial respiratory phosphorylation

in *iTJC1414x4* were assessed in separate in the model file “photosynthesis_and_mitochondrial_ATP.txt” (Supplementary File 4).

The minimal model combines six reactions of *iTJC1414x4* which represent the linear and cyclic photosynthetic electron transports as well as ATP synthesis (photophosphorylation). Four reactions representing the *iTJC1414x4* mitochondrial electron transport chain (complexes I, III and IV) and ATP production (complex V) were added as well. Mitochondrial oxidative phosphorylation with NADH oxidation via complex I is assumed to operate at a ATP/oxygen ratio of 3.33 (Lambers, 1997). We used Elementary Flux Modes analysis to obtain all possible stoichiometries for the net production ATP or NADPH per absorbed photon. Three elementary flux modes are obtained. In mode 1 (Table SF2-5), only CET takes place and photophosphorylation produces ATP at 0.5 ATP / photon. Mode 2 represents the transfer of photosynthetic reducing equivalents (LET) back to oxygen in mitochondria and ATP is produced via oxidative phosphorylation. The overall process produces no NADPH and 1.208 ATP per photon are obtained, which is more than twice as efficient than ATP production by CET. In mode 3, NADPH and ATP are produced via LET and photophosphorylation (0.25 NADPH and 0.375 ATP per photon absorbed), *i.e.* ATP and NADPH are produced at a ratio of 1.5. By linear combinations between modes one or two with mode 3, the ratio ATP / NADPH can be above 1.5.

Table SF2-5: Photosynthetic energy cofactor production in *iTJC1414x4*. Elementary flux modes of the combined chloroplast and mitochondrial electron transport chains and proton gradient-driven ATP generation are listed. This calculation is based on absorbed photons.

mode	description	net conversion
1	Photosynthesis, CET	2 photon + 1 ADP + 1 P_i + 1 H^+ = 1 H_2O + 1 ATP
2	Photosynthesis, LET & mitochondrial respiratory chain	24 photon + 29 ADP + 29 P_i + 29 H^+ = 29 H_2O + 29 ATP
3	Photosynthesis, LET	8 photon + 2 NADP + 3 ADP + 3 P_i + H^+ = O_2 + H_2O + 2 NADPH + 3 ATP

In the above section on photosynthesis stoichiometries, photons are understood as “absorbed” irradiation. However, the model was set up to predict light fluxes as “incident” instead of “absorbed” irradiation in order to compute quantum yields based in incident irradiation as done e.g. by Yin and Struik (2018). For this purpose, we considered electron transport efficiencies of Photosystems I and II as outlined by Yin and Struik (2018). Equations 7 to 10 in supporting information of Yin and Struik (2018) link incident irradiance to electron transport rates based on photosystem quantum yields Φ_{1LL} and Φ_{2LL}. Φ_{1LL}, for example, is defined as “Quantum yield of PSI photochemistry under limiting light [mol electron (mol photon)$^{-1}$]” and is assumed to be constant as long as the system doesn’t operate in saturated conditions. We use values reported in Table I of Yin and Struik (Φ_{2LL} = 0.8, Φ_{2LL}/Φ_{1LL} = 0.85; i.e. Φ_{1LL} =0.941176). The adjustments for photosystem inefficiencies for *iTJC1414x4* are as follows. In their original form, the stoichiometric equations for PSI and PSII in *iTJC1414x4* (‘*PhotosystemImodchloroplast*’, ‘*PhotosystemIImodchloroplast*’) represent that one electron is being moved per photon absorbed. To adjust for incident irradiation, the coefficients for photons are divided by the respective photosystem quantum yields. Accordingly, coefficient “4” for photons in ‘*PhotosystemIImodchloroplast*’ changed from 4 to 5 and coefficient for photons in ‘*PhotosystemImodchloroplast*’ changed from 1 to 1.0625. The adjusted reactions, with “**X**” indicating cell type BS or M, are:

X*PhotosystemImodchloroplast*:

1.0625 X*LightChloroplast*[**X**p] + 1 **X***OxidizedFerredoxinsChloroplast*[**X**p] + 1 **X***PlastocyaninReducedChloroplast*[**X**p] → 1 **X***OxidizedPlastocyaninsChloroplast*[**X**p] + 1 **X***ReducedFerredoxinsChloroplast*[**X**p]

X*PhotosystemIImodchloroplast*:
5 X*LightChloroplast*[**X**p] + 2 **X***PlastoquinoneChloroplast*[**X**p] + 4 **X***ProtonChloroplast*[**X**p] + 2 **X***WaterChloroplast*[**X**p] → **X***OxygenChloroplast*[**X**p] + 2 **X***Plastoquinol1Chloroplast*[**X**p] + 4 **X***ProtonThylakoidLumen*[**X**t]

(**X** = '*bsDay*', '*msDay*')

If we integrate Φ_{1LL} and Φ_{2LL} into the flux modes analysis ("photosynthesis_and_mitochondrial_ATP.txt"), then mode 1 (Table SF2-5a) produces ATP at 8/17 = 0.471 ATP/photon. Mode 2 produces ATP at 116/111 = 1.05 ATP/photon. Mode 3, LET, produces ATP at 12/37 = 0.324 ATP/photon (Table SF2-5a).

Table SF2-5a: Photosynthetic energy cofactor production in *iTJC1414x4*. Elementary flux modes of the combined chloroplast and mitochondrial electron transport chains and proton gradient driven ATP generation are listed. This calculation is based on "incident" photons.

mode	description	net conversion
1a	Photosynthesis, CET	17 photon + 8 ADP + 8 P_i + 8 H^+ = 8 H_2O + 8 ATP
2a	Photosynthesis, LET & mitochondrial respiratory chain	111 photon + 116 ADP + 116 P_i + 116 H^+ = 116 H_2O + 116 ATP
3a	Photosynthesis, LET	37 photon + 8 NADP + 12 ADP + 12 P_i + 4 H^+ = 4 O_2 + 4 H_2O + 8 NADPH + 12 ATP

3.2 Constraining linear electron transport (LET) relative to cyclic electron transport (CET) in *iTJC1414x4*

The model by Yin and Struik (2018) considers that the absorbed light drives LET and CET at proportions that depend on the composition of the photosynthetic apparatus. They determined that for sorghum, the fraction of absorbed light that drives LET (f_{LET}) was 0.9 for M cells and 0.03 for BS cells. In the following we derive the equation that constrains photosynthetic electron transport in *iTJC1414x4* to f_{LET}. To apply f_{LET} to our model, we have to consider the stoichiometries of photosystem I (*PSI*, reaction 'PhotosystemImodchloroplast') and photosystem I (*PSII*, reaction 'PhotosystemIImodchloroplast'), which are reproduced here in a compact form:

PSI: **1.0625** photon + 1 PC_{red} + 1 Fd_{ox} = 1 PC_{ox} + 1 Fd_{red} (1 electron moved per 1.0625 absorbed photon)
PSII: **5** photon + 2 H_2O + 2 PQ = O_2 + 2 PQH_2 (4 electrons moved per 5 absorbed photons)

Let v_{PSI} and v_{PSII} be the rates for the above reactions *PSI* and *PSII*, respectively. We are seeking the linear relation between v_{PSI} and v_{PSII} that depends on f_{LET}. The sum of photons absorbed by both photosystems is:

$$\text{total absorbed photons} = \mathbf{1.0625}\cdot v_{PSI} + \mathbf{5}\cdot v_{PSII}. \quad (3.1)$$

As a special case, if only LET takes place, electrons pass though PSI and PSII at the same rate. Due to above reaction equations this means that the reaction rate of *PSI* must be four times that of *PSII*:

$$v_{PSI} = 4 \cdot v_{PSII}. \quad (3.2)$$

Accordingly, we replace "v_{PSI}" in equation (3.1) with "$4 \cdot v_{PSII}$" and obtain:

$$absorbed\ photons_{(only\ LET)} = \mathbf{1.0625} \cdot 4 \cdot v_{PSII} + \mathbf{5} \cdot v_{PSII} = 9.25 \cdot v_{PSII}\ . \quad (3.3)$$

Using equations 3.1 and 3.3. we can write the fraction of absorbed light that drives LET (f_{LET}) as:

$$f_{LET} = \{absorbed\ photons_{(only\ LET)}\} / \{total\ absorbed\ photons\} = (9.25 \cdot v_{PSII}) / (5 \cdot v_{PSII} + 1.0625\ v_{PSI}),$$

which can be rearranged to:

$$v_{PSI} \cdot 1.0625 \cdot f_{LET} + v_{PSII} \cdot (5 \cdot f_{LET} - 9.25) = 0\ . \quad (3.4)$$

Equation (3.4) is applied as a numerical constraint to the day-time BS- and M sub-models by use of the COBRA function '*addCOBRAConstraints*'.

3.3 Deriving sorghum-specific values for energy budget parameters ($a_{BS,M}$, $f_{LET,BS}$, $f_{LET,M}$) from the Yin and Struik (2018) model

Model code for the "model for cell-type specific electron transport of C_4 photosynthesis" was taken from supplement of Yin and Struik (2018) (https://nph.onlinelibrary.wiley.com/action/downloadSupplement?doi=10.1111%2Fnph.15051&file=nph15051-sup-0001-SupInfo.pdf) and converted to code that can be executed in MatLab (m-code, see file "YinStruikModel.m"). The code contains definitions of various input parameters. Input constants that determine light absorption and distribution are set to specify sorghum. Other constants that define the biosynthetic demands can be modified. The equations and all variables are explained in detail in the supplement of Yin and Struik (2018).

To have some confidence that the code was correctly adapted, we reproduce sorghum specific results reported in the publication by using the following settings in the m-code file:

`Phi = 0.16; VOC = 0; VNC = 0; RDC = 0; STARCH = 0;` (Case A in below table)

- or -

`Phi = 0.16; VOC = 0.05; VNC = 0.0286; RDC = 0.025; STARCH = 1/6;` (Case B in below table).

The results are compared with Table 2 in Yin and Struik (2018) below:

		Reproduced values		Values reported in Table 2 (column NADPH-ME, *Sorghum bicolor*)	
**Variable**	**Table 2, outputs,**	**A**	**B**	**A**	**B**
`Absm`	line 1	0.3984	0.3984	0.40	0.40
`CxT`	line 2	0.8073	0.6862	0.81	0.69
`BETA`	line 3	0.7491	0.8159	0.75	0.82
`PSbsm`	line 4	0.3064	0.2954	0.31	0.30
`KbsKm`	line 5	1.3005	1.3487	1.30	1.35
`PS12`	line 6	1.6573	1.5362	1.66	1.54

PS12BS	line 7	61.3186	56.8386	61.32	56.84
PS12M	line 8	1.0546	0.9776	1.05	0.98
U	line 9	0.9004	0.9355	0.90	0.94
V	line 10	0.0297	0.0320	0.03	0.03
FCET	line 11	0.5370	0.5082	0.54	0.51
FCETBS	line 12	0.9861	0.9850	0.99	0.99
FCETM	line 13	0.1940	0.1305	0.19	0.13
ReBSM	line 14	0.7638	0.7921	0.76	0.79
RcetBS	line 15	0.7952	0.8567	0.80	0.86
RNADPHBS	line 16	0.0130	0.0134	0.01	0.01
RATPBS	line 17	0.3541	0.3574	0.35	0.36
RaCBS	line 18	0.9794	0.9777	0.98	0.98
RATPLET	line 19	0.5639	0.5921	0.56	0.59
FCO2	line 20	0.0705	0.0654	0.071	0.065

A, only cost for CBB cycle are considered. No photorespiration, alternative electron, nor ATP sinks;
B, photorespiration, alternative electron, and ATP sinks are considered.

We next define modified parameters to be used as inputs for *iTJC1414x4*. We set the leakiness parameter (`Phi`) to zero, the constants for photorespiration other sinks (`VOC, VNC, RDC`) to zero and set ATP cost for starch synthesis to 1/12 ATP per CO_2 fixed, which is the cost to make sucrose from triose phosphate in *iTJC1414x4* (1 ATP per 12 CO_2 fixed into one sucrose):

```
Phi = 0; VOC = 0; VNC = 0; RDC = 0; STARCH  = 1/12;
```

Among the outputs generated by the code we used the energy budget parameters (`Absm, U, V`), here referred to as ($a_{BS,M}$, $f_{LET,M}$, $f_{LET,BS}$). Another output we use is `fCO2`, the quantum yield of CO_2 assimilation on the absorbed light basis.

Results:

```
Absm = 0.398435839172528
U = 0.933101822698474
V = 0.0318234752990049
fCO2 = 0.0731150038080731
```

Above values for `Absm`, `U` and `V` were used as inputs ($a_{BS,M}$, $f_{LET,M}$, $f_{LET,BS}$) for our model. The model was configured for CO_2 assimilation into sucrose, with photorespiration, leakiness and maintenance energy burdens inactivated. Then the model produces a flux state with balanced energy budget for sucrose synthesis with a quantum yield of 0.073 (Main text, Table 3, scenario 2, sucrose synthesis).

Next, we tried to find a flux state with balanced energy budget for sucrose synthesis when *iTJC1414x4* is configured with active photorespiration, CO_2 leakage and maintenance energy burdens. We could not use the input constants of the Yin and Struik model to create such a scenario. Instead, the state above ($a_{BS,M}$ = 0.398, $f_{LET,M}$ = 0.9331, $f_{LET,BS}$ = 0.0318) was used as a starting point and then $f_{LET,M}$ was adjusted in small steps until an energy balanced state in *iTJC1414x4* was reached. The value for $f_{LET,M}$ was 0.8752122.

3.4 Calculations for light-driven production of reducing equivalents and ATP from model simulations in *iTJC1414x4*

For various simulations of photosynthetic conversion of CO_2 into a product the supply in ATP and reducing equivalents (NADPH) by photosynthetic light reactions was calculated based on model reaction rates and then expressed relative to the amount of CO_2 assimilated. We consider here the mitochondrial electron transport coupled with ATP production as part of the supply side (Fig 2B, main text). Reducing power can be transferred from the chloroplast into the mitochondrion by reactions the "malate valve" shuttle system (Selinski and Scheibe, 2019). The calculations used to determine ATP and NADPH supply are as follows ("**X**" stands for cell type BS or M):

NADPH/CO_2 assimilated: The number of reducing equivalents produced per CO_2 assimilated into sucrose or TAG was determined as:

$$H_2biosynthesis\mathbf{X} = (H_2PSET\mathbf{X} - H_2MET\mathbf{X})/CO_2NET$$

with

$$CO_2NET = \textit{msDaytxCARBONDIOXIDE}$$

being the net CO_2 uptake from the environment that is being used in sucrose or TAG biosynthesis. H_2PSET is the number of reducing equivalents produced from photosynthetic electron transport, derived from the chloroplast photosystem II stoichiometry. Corrections for electrons going back to oxygen through Mehler reaction and superoxide dismutase are included (although these were generally inactive in flux predictions):

$H_2PSET\mathbf{X}$ = 2*(**X***DayPhotosystemIImodchloroplast* - **X***DayMehlerReactionchloroplast* + **X***DaySUPEROXDISMUTRXNchloroplast*)

$H_2MET\mathbf{X}$ is the number of reducing equivalents transferred onto oxygen through mitochondrial electron transport, derived from the mitochondrial complex IV stoichiometry:

$$H_2MET\mathbf{X} = 2*\mathbf{X}\textit{DayComplexIVmodmitochondrion}$$

The term '- $H_2MET\mathbf{X}$' is used to account for reducing equivalents that are transferred back to oxygen in mitochondria. In our model the complex IV of the mitochondrial electron transport activity is fully linked to complex V, i.e. there are no alternative pathways that discharge electrons without producing ATP. Such pathways such as alternative oxidase would most likely be suboptimal under typical FBA simulation conditions. All the reducing equivalents that enter the mitochondrial electron transport chain and oxidative phosphorylation originate in some way from photosystem, which is also represented by flux mode 2 in Table SF2-5. Therefore, after the correction by '- $H_2MET\mathbf{X}$', '$H_2biosynthesis\mathbf{X}$' represents the rate of production of reducing equivalents that could be available to biosynthetic activities.

ATP/CO_2 assimilated: ATP generation for use in sucrose or TAG synthesis (per CO_2 assimilated) is quantified as:

$$\textit{ATP\_biosynthesis}\mathbf{X} = (ATP\_PS\mathbf{X} + ATP\_mit\mathbf{X})/CO_2NET$$

with ATP_PS**X** being ATP produced from chloroplast proton coupled ATPase (stoichiometry is 1 ATP per 3 protons translocated):

ATP_PS**X** = 3***X***DayChloroplasticATPasemodchloroplast*

and ATP_mit**X** being the ATP produced from mitochondrial proton coupled ATPase (stoichiometry is 1 ATP per 3 protons translocated):

ATP_mit_**X** = **X***DayComplexVmodmitochondrion*

Supplemental References

Battley EH (1999) An empirical method for estimating the entropy of formation and the absolute entropy of dried microbial biomass for use in studies on the thermodynamics of microbial growth. Thermochimica Acta **326:** 7-15

Bauwe H, Hagemann M, Fernie AR (2010) Photorespiration: players, partners and origin. Trends Plant Sci **15:** 330-336

Chan DI, Vogel HJ (2010) Current understanding of fatty acid biosynthesis and the acyl carrier protein. Biochem J **430:** 1-19

Hanson RW (1989) The Role of ATP in Metabolism. Biochemical Education **17:** 86-92

Joyard J, Ferro M, Masselon C, Seigneurin-Berny D, Salvi D, Garin J, Rolland N (2010) Chloroplast proteomics highlights the subcellular compartmentation of lipid metabolism. Prog Lipid Res **49:** 128-158

Jurowski K, Krzeczkowska MK, Jurowska A (2015) Approaches to Determining the Oxidation State of Nitrogen and Carbon Atoms in Organic Compounds for High School Students. Journal of Chemical Education **92:** 1645-1652

Kruger NJ (1997) Carbohydrate synthesis and degradation. *In* DT Dennis, DH Turpin, DD Lefebvre, DB Layzell, eds, Plant Metabolism. Addison Wesley Longman, Harlow, pp 83-104

Lambers H (1997) Oxidation of mitochondrial NADH and the synthesis of ATP. *In* DT Dennis, DH Turpin, DD Lefebvre, DB Layzell, eds, Plant Metabolism. Addison Wesley Longman, Harlow, pp 200-219

Li-Beisson Y, Shorrosh B, Beisson F, Andersson MX, Arondel V, Bates PD, Baud S, Bird D, Debono A, Durrett TP, Franke RB, Graham IA, Katayama K, Kelly AA, Larson T, Markham JE, Miquel M, Molina I, Nishida I, Rowland O, Samuels L, Schmid KM, Wada H, Welti R, Xu C, Zallot R, Ohlrogge J (2013) Acyl-lipid metabolism. Arabidopsis Book **11:** e0161

Noctor G, Foyer CH (1998) A re-evaluation of the ATP :NADPH budget during C3 photosynthesis: a contribution from nitrate assimilation and its associated respiratory activity? J Exp Bot **49:** 1895-1908

Pastore D, Di Pede S, Passarella S (2003) Isolated durum wheat and potato cell mitochondria oxidize externally added NADH mostly via the malate/oxaloacetate shuttle with a rate that depends on the carrier-mediated transport. Plant Physiol **133:** 2029-2039

Peterhansel C, Horst I, Niessen M, Blume C, Kebeish R, Kurkcuoglu S, Kreuzaler F (2010) Photorespiration. Arabidopsis Book 8: e0130

Pfeiffer T, Sánchez-Valdenebro I, Nuño JC, Montero F, Schuster S (1999) METATOOL: for studying metabolic networks. Bioinformatics **15:** 251-257

Ritchie R (2010) Modelling photosynthetic photon flux density and maximum potential gross photosynthesis. Photosynthetica **48:** 596-609

Schmidt-Rohr K (2015) Why Combustions Are Always Exothermic, Yielding About 418 kJ per Mole of O2. Journal of Chemical Education **92:** 2094-2099

Schuster S, Dandekar T, Fell DA (1999) Detection of elementary flux modes in biochemical networks: a promising tool for pathway analysis and metabolic engineering. Trends Biotechnol **17:** 53-60

Schuster S, Fell DA, Dandekar T (2000) A general definition of metabolic pathways useful for systematic organization and analysis of complex metabolic networks. Nature Biotechnology **18:** 326-332

Selinski J, Scheibe R (2019) Malate valves: old shuttles with new perspectives. Plant Biol (Stuttg) **21 Suppl 1:** 21-30

Shanklin J, Cahoon EB (1998) Desaturation and related modifications of fatty acids. Annual Review of Plant Physiology and Plant Molecular Biology **49**: 611-641

Sharkey TD, Weise SE (2012) Autotrophic Carbon Dioxide Fixation. In JJ Eaton-Rye, BC Tripathy, TD Sharkey, eds, Photosynthesis: Plastid Biology, Energy Conversion and Carbon Assimilation. Springer Netherlands, Dordrecht, pp 651-674. DOI: 10.1007/978-94-007-1579-0_26

Stitt M, Huber S, Kerr P (1987) 6 - Control of Photosynthetic Sucrose Formation. *In* MD Hatch, NK Boardman, eds, Photosynthesis. Academic Press, pp 327-409

Yin X, Struik PC (2018) The energy budget in C4 photosynthesis: insights from a cell-type-specific electron transport model. New Phytol **218:** 986-998
