# Supplementary material for: Elucidation of Triacylglycerol Overproduction in the C4 Bioenergy Crop Sorghum bicolor by Constraint-Based Analysis
Source: Front Plant Sci. 2022 Feb 17;13:787265. doi: 10.3389/fpls.2022.787265 (PMC8892208; doi:10.3389/fpls.2022.787265)
Supplement: Supplementary file 5 [file Image_1.pdf]

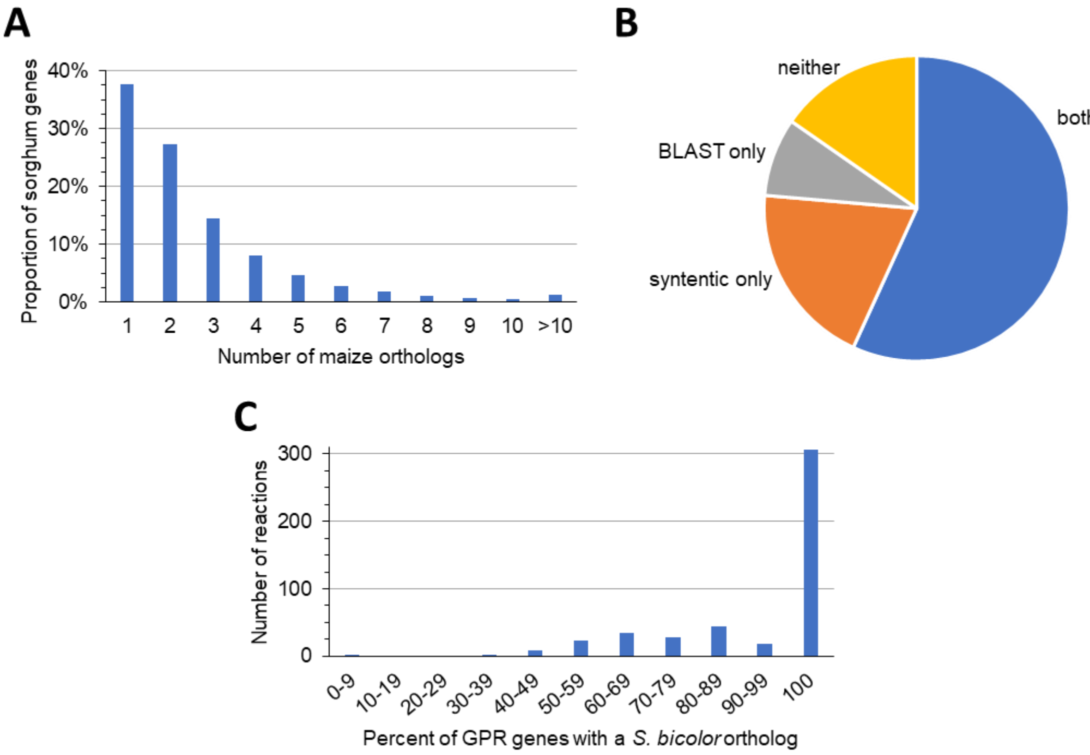

Supplementary Figure 1 | Maize to sorghum ortholog gene mapping. Syntenic orthology relations between protein encoding chromosomal genes of *Zea mays* B73 v3 and *Sorghum bicolor* v3.1.1 were derived with the SynOrths tool. Independent of the SynOrths tool, additional homology relationships were established by protein BLAST alignments. Homology was given for at least 70% identity in amino acids with at least 90% of the sorghum protein sequence in alignment. **(A)** Number of maize orthologs per sorghum gene locus. **(B)** Percentage of *iEB2140* maize genes identified by syntentic orthology analysis (red), additional independent BLAST alignment (gray), or both (blue). Yellow indicates the fraction of genes for which no orthology or homology relation was found. **(C)** The frequency of sorghum orthologs identified for reactions in *iEB2140*. Plotted are the number of reactions that were found to have orthologs for the indicated proportion of associated genes.

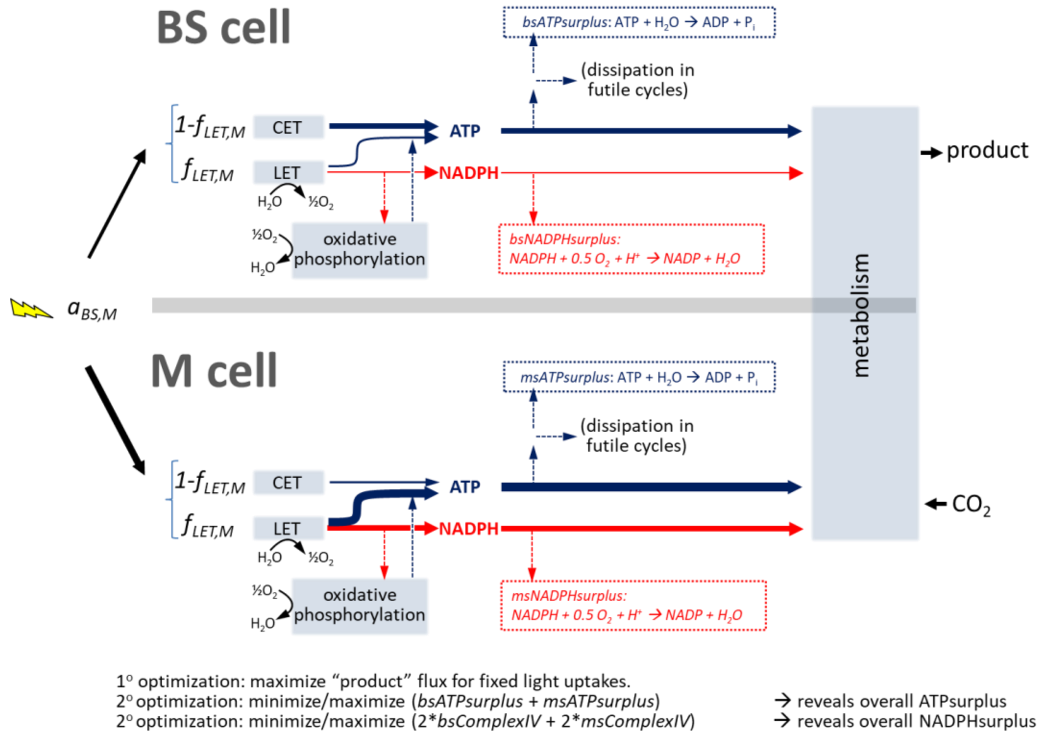

Supplementary Figure 2 | Concept of assessing the supply/demand balance in *iTJC1414x4* by Flux Balance Analysis (FBA) if energy budget parameters $a_{BS,M}$, $f_{LET,BS}$ and $f_{LET,M}$ are set. The parameter f_{LET} fixes the proportions of absorbed light energy that drive linear electron transport (LET) and cyclic electron transport (CET). This, in turn, in each cell, sets the ratio of photosynthetically produced ATP and NADPH to a fixed ratio. In a series of scenarios, the energy balance was explored (Table S7). Considering light limited optimal solution states, the photosynthetic energy budget is balanced if the supply ratio and the demand ratio agree in both sub-models. If too much ATP is produced, then the surplus will be consumed in the reaction network by ATP wasting activities like ATP consuming futile cycles. The FBA solution space typically allows a wide range of alternative solutions to dissipate surplus ATP in the network. The totality of the ATP surplus can be quantified by a serial minimization and maximization procedure of the reaction *ATPsurplus* as indicated. Both minimization and maximization will give the same result, i.e., the ATP surplus is invariable in the FBA solution space. In order to detect overproduction of NADPH by LET, it would appear that maximization of the reaction *NADPHsurplus* allows quantifying the NADPH surplus in an analogous way. However, we observed that optimal flux solutions will obtain an adjustment of supply and demand via mitochondrial oxidative phosphorylation. The NADPH surplus is identified by a serial minimization and maximization procedure of the complex IV reaction representing mitochondrial electron transport.

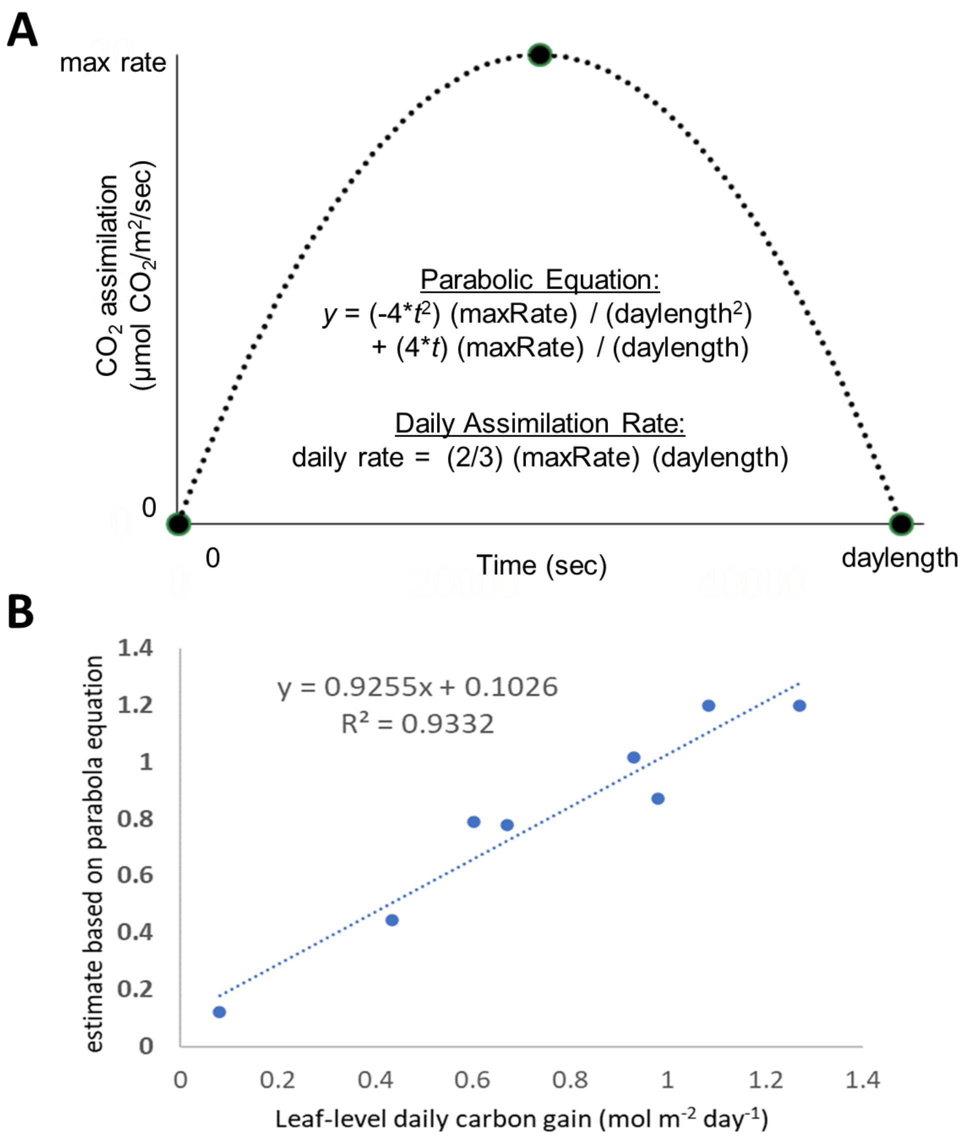

Supplementary Figure 3 | Estimation of daily carbon assimilation. (A) The rate of carbon fixation was assumed to show parabolic variation throughout the day, where the parabolic equation intercepts the *x*-axis at sunrise and sunset and peaks at a rate not limited by light availability ("maxRate"). The daily assimilation rate could then be approximated by integrating the parabola over the daylength. Equations for this parabola and its integration are shown. **(B)** The use of the parabola approximation was validated using experimental data from Dohleman et al., (2009). x-axis: leaf-level daily carbon gain taken from Figure 3 of Dohleman et al., (2009) (miscanthus, year 2006 data). The authors derived these as integrals of the diurnal course of net photosynthetic leaf CO_2 uptake ("Daily integrated rates of leaf CO_2 uptake (A′) were calculated from the instantaneous measurements made for each day of the growing season by summing the trapezoidal area described under each pair of adjacent measurement times, when plotted against time of day."). Y-axis: The parabola equation was applied to daily time course data in Dohleman et al., (2009) (Figure 2b, miscanthus, year 2006). Readouts for daylength and maximal photosynthetic rate of were transformed with the parabolic equation to daily carbon gain. Linear fit was done using the linear trendline function in Microsoft Excel.

Dohleman FG, Heaton EA, Leakey ADB, Long SP (2009) Does greater leaf-level photosynthesis explain the larger solar energy conversion efficiency of Miscanthus relative to switchgrass? Plant Cell Environ 32: 1525-1537 https://doi.org/10.1111/j.1365-3040.2009.02017.x

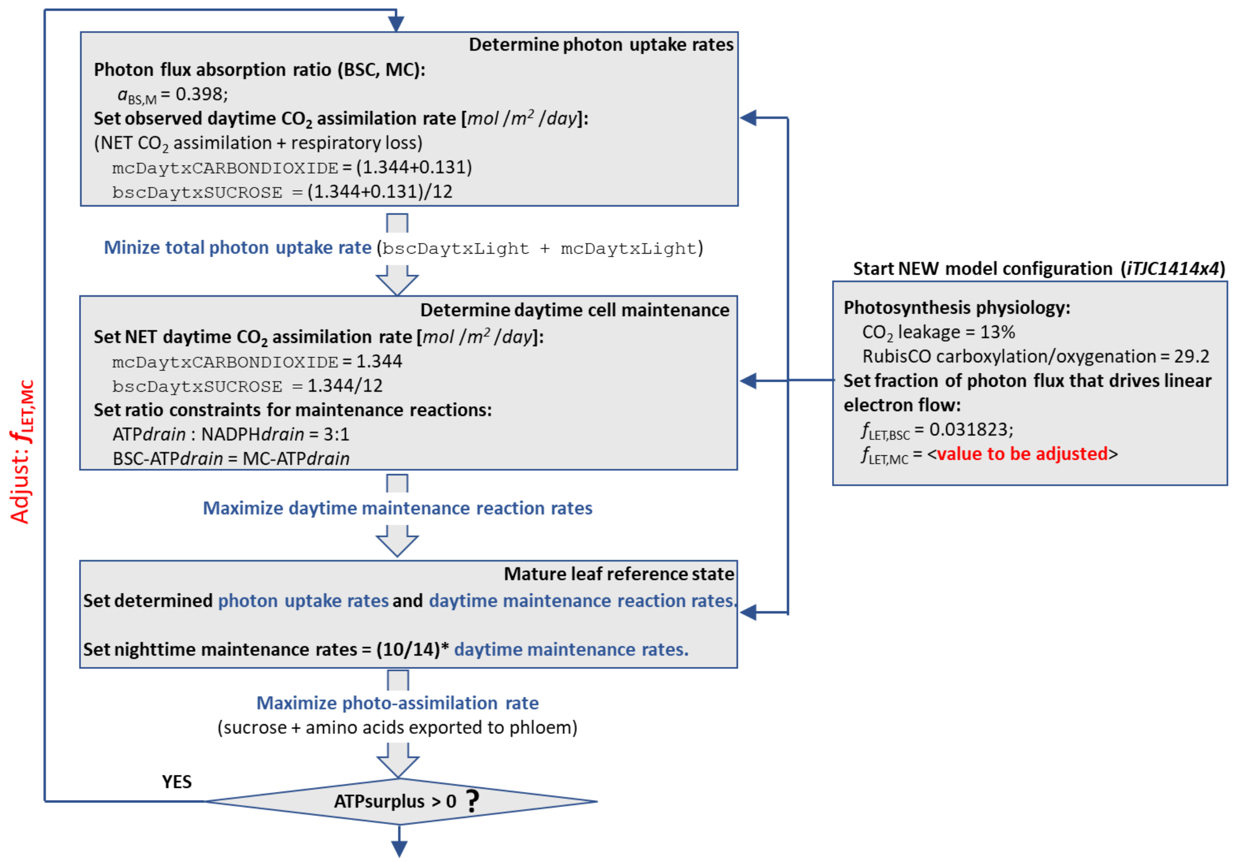

Supplementary Figure 4 | Procedure to generate a mature leaf reference state for the diel model (*iTJC1414x4*). Photon uptake rates and maintenance fluxes are determined based on empirical physiological data. The procedure starts with energy budget parameters ($a_{BS,M}$, $f_{LET,BS}$, $f_{LET,M}$) from the Yin and Struik (2018) model for sorghum leaf photo-assimilation of CO_2 into sucrose, without consideration of other metabolic burdens such as cellular maintenance or nitrate assimilation. Linear electron flow in M cells is adjusted until the energy budget is balanced (ATP surplus = 0) for production of mixed photo-assimilate (sucrose and amino acids, Supplementary Table 8). Nighttime maintenance is set based on a daytime:nighttime ratio of 14h:10h. Flux units are $mol\ m^{-2}\ day^{-1}$.

Abbreviations: $a_{BS,M}$, ratio of day model photon uptakes into BS and M cells (reactions in *iTJC1414x4*: '*bscDaytxLight*', '*msDaytxLight*'; see equation 6 in main text); ATP*drain*, generic ATPase ('*bscDayGenericATPasemod*', '*msDayGenericATPasemod*'); $f_{LET,BS}$, $f_{LET,M}$, fraction of absorbed light in BS/M cells that drives linear electron transport; NADPH*drain*, generic NADPH oxidizing reactions ('*bscDayGenericNADPOxidasemod*', '*mcDayGenericNADPOxidasemod*').

	RELATIVE DEPOSITION RATES (%)			
**MAXIMIZED TAG ACCUMULATION RATES:**	***Day, BSC***	***Day, MC***	***Night, BSC***	***Night, MC***
bscDay + mcDay + bscNight + mcNight	[0-100]	[0-100]	[0-97.8]	[0-97.8]
bscDay + mcDay	[0-100]	[0-100]	n/a	n/a
bscDay	[0-100]	n/a	n/a	n/a
mcDay	n/a	[0-100]	n/a	n/a
bscNight + mcNight	n/a	n/a	[0-97.8]	[0-97.8]
bscNight	n/a	n/a	[0-97.8]	n/a
mcNight	n/a	n/a	n/a	[0-97.8]

Supplementary Figure 5 | Efficiency of TAG storage in sub-models of *iTJC1414x4*. Triacylglycerol (TAG) accumulation in *Sorghum bicolor* was modeled with *iTJC1414x4* set to the mature leaf reference state. In separate optimization scenarios, the TAG deposition reactions for all four sub-models (*mcDayOilDroplet*, *bscDayOilDroplet*, *mcNightOilDroplet*, *bscNightOilDroplet*) were maximized in combinations as indicated. Subsequent optimizations then minimized / maximized values for individual TAG deposition reactions. Shown are the resulting relative flux rates, normalized by the overall maximum TAG flux rate. The 100% value is 0.013569 mol m^{-2} day^{-1}. If present, flux variability intervals are given ([*lower bound*, *upper bound*]).

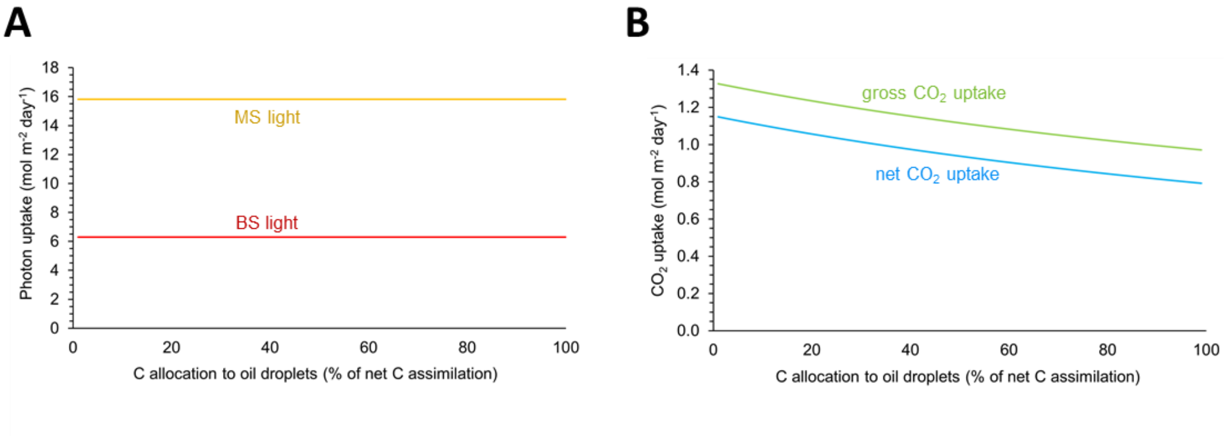

Supplementary Figure 6 | Variation in uptake rates in response to percent carbon allocation. Light and CO_2 uptake were constrained in *iTJC1414x4* to be consistent with *Sorghum bicolor* leaf physiology (see Results). As net carbon allocation toward triacylglycerol (TAG) increased, the **(A)** light uptake per mesophyll cell (MC) and bundle sheath cell (BSC) remained constant, while **(B)** gross and net CO_2 uptake in the M cell decreased.

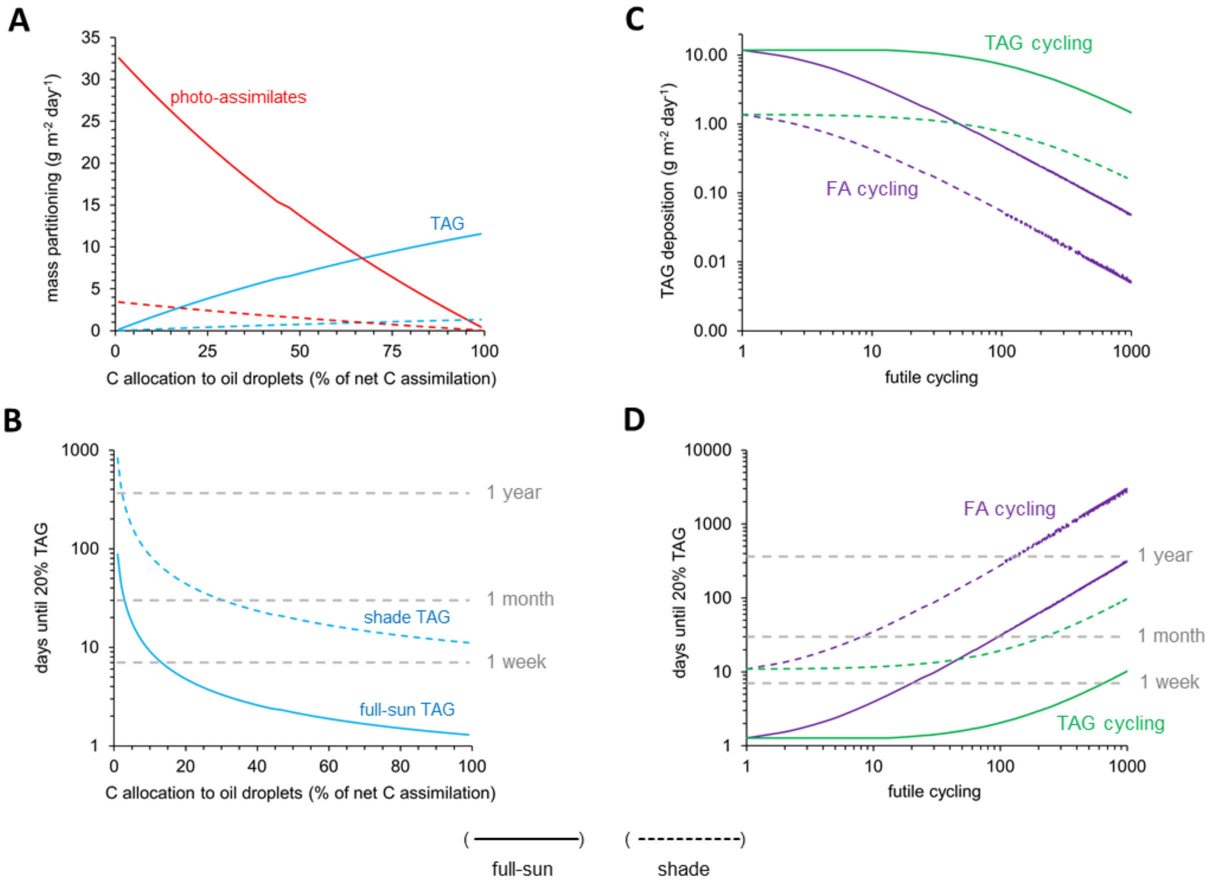

Supplementary Figure 7 | Comparing model simulations for TAG accumulation in full-sun and shaded leaves. Daily TAG accumulation was modeled (*iTJC1414x4*) for mature *Sorghum bicolor* leaves exposed to full sun (solid lines) or when light availability was 70% shaded (dotted lines). See Supplemental Table S1 for model constraints. Plotted are the **(A,C)** triacylglycerol (TAG) deposition rate and **(B,D)** time-period until 20% (w/dw) TAG accumulation is reached in dependence on the fraction of assimilated carbon being allocated to TAG (purple) and the degrees of TAG (blue) or fatty acid (FA; green) futile cycling.
